# Assessment of Transfer Learning for H&E Virtual Staining in Veterinary Oncology

**DOI:** 10.3390/jimaging12100490

**Published:** 2026-10-06

**Authors:** Daira Viškere, Diāna Dupļevska, Ilze Matīse-VanHoutana, Romans Maļiks, Blaž Cugmas, Mindaugas Tamošiūnas, Roberts Kadiķis

**Affiliations:** 1 Biophotonics Laboratory, Institute of Atomic Physics and Spectroscopy, Faculty of Science and Technology, University of Latvia, 19 Raina Blvd., LV-1586 Riga, Latvia; imatise.vh@gmail.com (I.M.-V.); blaz.cugmas@lu.lv (B.C.); mindaugas.tamosiunas@lu.lv (M.T.); 2 Faculty of Veterinary Medicine, Latvia University of Life Sciences and Technologies, 8 K. Helmana Str., LV-3004 Jelgava, Latvia; 3 Institute of Electronics and Computer Science, 14 Dzerbenes Str., LV-1006 Riga, Latvia; diana.duplevska@edi.lv (D.D.); romans.maliks@edi.lv (R.M.)

**Keywords:** virtual staining, transfer learning, veterinary oncology, autofluorescence, hematoxylin and eosin, Pix2Pix dense neural network

## Abstract

Conventional hematoxylin and eosin staining is indispensable for histopathology but is time-, labor-, and resource-intensive, environmentally unsustainable, and sensitive to operator and reagent variability. We developed a two-stage transfer-learning virtual staining (VS) pipeline for generating H&E-like images from label-free autofluorescence (AF) slides. In Stage 1, a Pix2Pix Dense U-Net was trained under paired supervision using spatially registered fluorescence images of H&E-stained sections and brightfield H&E images acquired from the same physical sections. The fluorescence inputs contained channel-dependent eosin emission and hematoxylin-associated attenuation, which provided image contrast available to the network but were not encoded as explicit loss terms. In Stage 2, the retained Pix2Pix(v2) checkpoint initialized channel-specific Pix2Pix(v3) models, which were fine-tuned on registered single-channel fluorescence–H&E pairs (DAPI, Cy3, or FITC input to the corresponding full H&E target) and then applied to native AF images from unstained sections lacking paired H&E targets. The pipeline integrates HSV masking, ORB–RANSAC registration, multi-resolution tiling, SSCOR-based stripe suppression, a fine-tuned Pix2Pix Dense U-Net, and an evaluated CycleGAN color-normalization step that was ultimately discarded because it degraded SSIM/FID and expert scores. In a four-case pilot comparison, grayscale DAPI yielded the highest overall pathology-based scores and the most coherent whole-slide VS among the tested configurations; Cy3 and FITC were prone to discontinuities and hue shifts, and multi-channel composites underperformed. In a veterinary cohort (13 soft tissue sarcomas, 12 mast cell tumors), single-observer semiquantitative pathology scoring (technical quality, H&E staining, image quality, accuracy) found that tissue-level architecture and tumor margins were preserved, but cellular and nuclear detail was limited, precluding confident diagnosis or grading. Quantitatively, improved preprocessing and model refinement lowered FID and raised SSIM versus the tested baselines. This approach reuses archived H&E slides as source-domain supervision and provides a practical route for adapting virtual staining models to label-free AF data under real-world acquisition constraints.

## 1. Introduction

Histopathology uses hematoxylin and eosin (H&E) staining, which provides contrast for examining tissue architecture, nuclear morphology, and cytoplasmic features. Due to its high reliability and diagnostic value, H&E staining has become the standard method for diagnosing a wide range of diseases, including cancer, inflammatory conditions, infectious diseases, and other pathological abnormalities. However, the chemical process of H&E staining is not environmentally sustainable: it consumes time and energy via multiple tissue processing steps and its outcome also depends on staining conditions, chemical reagent quality, and operator expertise. Moreover, the staining procedure generates significant volumes of chemical and plastic waste and contaminates water during repeated rinsing steps. As healthcare systems increasingly adopt environmentally responsible practices, there is a growing search for sustainable staining alternatives that preserve the diagnostic efficacy of conventional H&E.

Virtual staining (VS) using artificial intelligence (AI)/deep learning has emerged as a viable solution for computational generation of histologically stained images from label-free tissue samples. This eliminates the need for traditional chemical staining procedures that are time-consuming, labor-intensive, costly, and environmentally impactful. The dominant deep learning approaches in virtual staining research are Pix2Pix [1], CycleGAN [2] and CUT [3]. Pix2Pix is a supervised paired image-to-image translation framework that requires aligned source–target examples. Paired virtual-staining research includes work by Rivenson [4]; related deep-learning components relevant to virtual-histology pipelines include work by Li and Wang [5,6,7]. CycleGAN provides unpaired image-to-image translation when aligned source–target pairs are unavailable. Related GAN-based processing and virtual-histopathology approaches include Wang [7], Cao [8], de Haan [9], Rana [10] and Nerrienet [11]. CUT (Contrastive Unpaired Translation) also does not require paired training samples; it uses a one-sided contrastive objective and avoids the bidirectional cycle-consistency formulation of CycleGAN. CUT and related contrastive unpaired methods have been used or adapted in histopathology and virtual-staining studies by Pati [12], Yoon [13] and Asaf [14]. Despite these advances, practical challenges remain: obtaining precisely registered image pairs, handling scanner-induced stripe artifacts, maintaining color consistency across slides, and limited validation on veterinary pathology samples.

Strong structural supervision arises from pixel-level correspondence between the unstained images and their matching H&E image pairs. This enables preservation of cellular-level morphology and tissue architecture, for example—correct distribution of cell nuclei and cytoplasm and accurate mapping of tumor-stroma boundaries. In supervised virtual staining workflows, optical contrast images—autofluorescence (AF) [4,15] or photoacoustic remote sensing [16] are usually acquired first from the unstained histopathology slide section, followed by H&E staining of the same slide section and reimaging it to generate the pixel-aligned pairs. Then the paired AF ↔ H&E data are used to train a neural network (NN) with the aim of producing virtual H&E contrast on the unseen optical images fed into the trained NN model.

Other VS approaches have relied on adjacent histopathology sections without exact pixel-level correspondence. For example, photoacoustic remote sensing/scattering microscopy images acquired from an unstained section have been compared with H&E-stained images from an adjacent serial section of the same tissue block [17]. Although these sections are not perfectly aligned, the generated virtual H&E images retained sufficient contrast and resolution to resolve nuclei, collagen-rich stroma, and vascular structures.

The unpaired training approaches offer additional value for histopathology; for example, Kang [18] demonstrated that photoacoustic microscopy images could be translated to H&E contrast without pixel alignment, allowing VS to be implemented even when paired data cannot be obtained. However, unpaired image-to-image translation is intrinsically under-constrained and can exhibit structural or chromatic artifacts; generated-image evaluation therefore benefits from both qualitative assessment and distributional metrics [5,10,19].

Prior work has shown that adding a small number of paired samples to otherwise unpaired training sets can improve the performance of unpaired image-to-image translation models by enhancing structural fidelity, stabilizing color mapping, reducing artifacts or hallucinated details [20,21] or by introducing a supervised refinement stage using registration-derived correspondences, as demonstrated by Biswas [22]. This highlights the value of additional structural supervision for preserving image-translation quality and maintaining morphology meaningfulness.

Unlike fully paired native AF→H&E approaches, our workflow separates supervised source-domain learning from target-domain inference. Stage 1 uses spatially registered fluorescence images of H&E-stained sections and brightfield H&E images from the same physical section to train the Pix2Pix Dense U-Net. In Stage 2, the retained Pix2Pix(v2) checkpoint initializes channel-specific Pix2Pix(v3) models, which are fine-tuned on registered DAPI–H&E, Cy3–H&E, and FITC–H&E channel-wise pairs. Although the H&E target is the full brightfield image rather than a channel-specific target, each input and target correspond to the same registered tissue region; thus, Stage 2 fine-tuning remains supervised. The resulting models are then applied to native, unstained AF data for which no paired H&E target is available. Accordingly, the unpaired data are used for inference rather than for optimization of Pix2Pix(v3).

**Figure 1:**
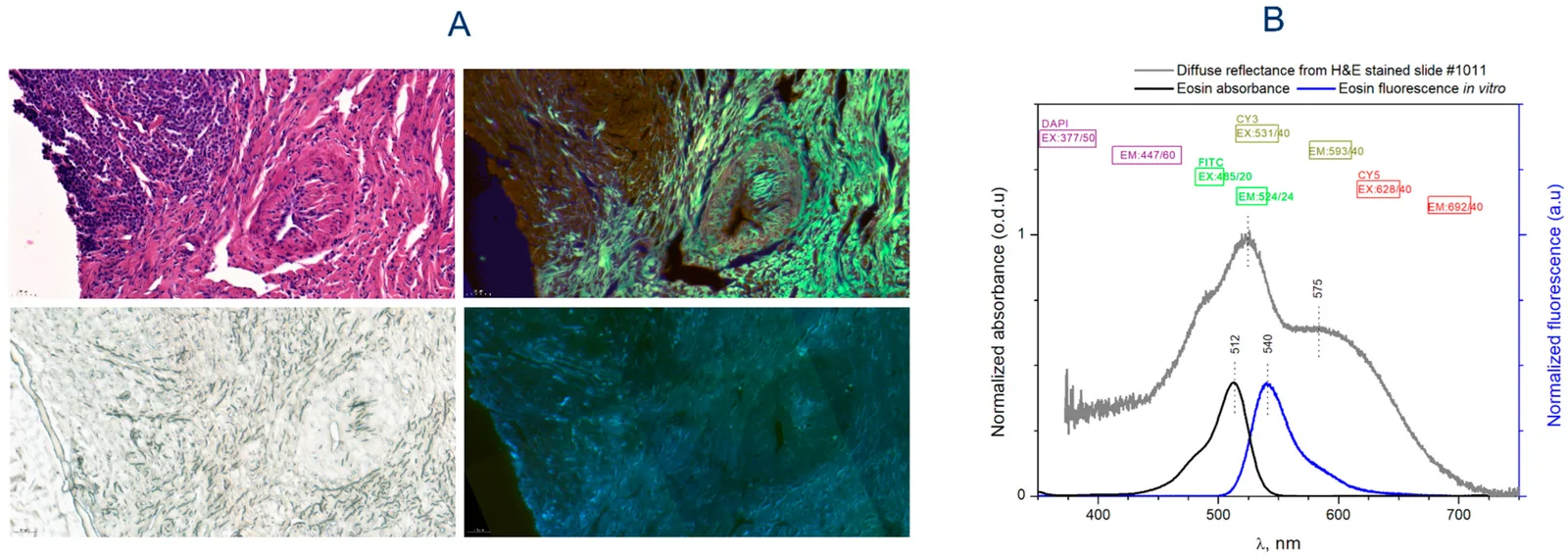
(**A**) Multispectral imaging data used for paired and unpaired virtual staining. Representative case #1011 of high malignancy grade mast cell tumor located between second and third right mammary gland of 15-year-old Poodle. Top-left: brightfield reference image of H&E stained section. Dense infiltrate of tumor mast cells is located at the top left corner. Adjacent to this, there is a large amount of collagen-rich fibrous stroma surrounding a blood vessel with thickened walls (oval structure, center of the image). Top-right: H&E stain enhanced multispectral fluorescence image (comprising inputs from DAPI, FITC, Cy3 and Cy5 channels), also illustrating eosin-driven structural emission and hematoxylin-associated attenuation. Bottom-left: Brightfield image of the adjacent/next unstained serial section. Bottom-right: Native autofluorescence comprising emission in DAPI, FITC, Cy3, Cy5 channels, used as a label-free input for transfer learning (TL) to generate H&E virtual staining. We highlight that even when the top-right and the bottom right images originate from the adjacent serial sections of the same tissue block, they do not exhibit direct pixel-level correspondence. A notable structural variation, contour change and slight spatial displacement of the central blood vessel is noticeable between these adjacent sections. However, the top-left and top-right images acquired from the same physical tissue section preserve spatial arrangement of tissue structures, and enable pixel-to-pixel correspondence suitable for supervised paired training. (**B**) Spectral behavior of H&E chromophores relative to the DAPI, FITC, Cy3 and Cy5 filter channels. Grey line: diffuse reflectance spectrum of the H&E-stained slide (case #1011); black line: normalized eosin absorbance; blue line: normalized eosin fluorescence emission, both measured *in vitro* in aqueous solution. Labelled boxes indicate the excitation (EX) and emission (EM) bands of each channel (centre wavelength/bandwidth, nm): DAPI EX 377/50, EM 447/60; FITC EX 485/20, EM 524/24; Cy3 EX 531/40, EM 593/40; Cy5 EX 628/40, EM 692/40. *In vitro*, eosin absorbance peaks at ~512 nm, whereas in tissue it is red-shifted to ~524 nm due to protein binding. The residual diffuse reflectance signal corresponds to hematoxylin absorbance, [23]. Eosin emission peaks at ~540 nm, mirroring its absorbance spectrum, which increases toward longer wavelengths and peaks near ~575 nm (within the Cy3–Cy5 excitation range), consistent with Gibbs’s [23] results. Eosin emission peaks near ~540 nm, mirroring its absorbance spectrum. Absorbance and diffuse reflectance spectra were measured as described in Maciulevičius [24].

To our knowledge, prior virtual H&E staining studies have not explicitly examined how the spectral properties of eosin fluorescence and hematoxylin-associated attenuation across DAPI, FITC, Cy3, and Cy5 relate to AF→H&E virtual staining. In this study, these measured optical properties were used to motivate the channel-specific analysis; they were not implemented as an explicit loss term or additional neural-network constraint. As shown in Figure 1B, eosin exhibits the strongest fluorescence emission in FITC and Cy3 channels, increasing cytoplasmic and stromal fluorescence signals above native AF levels. Hematoxylin is non-fluorescent and instead attenuates excitation light through absorption, with stronger attenuation in the Cy3–Cy5 spectral region and detectable effects in DAPI and FITC. These channel-dependent contrast patterns were present in the registered fluorescence images of the H&E-stained source sections and were therefore available to the network during supervised training, but they were not separately enforced by the optimization objective. The measured spectral behavior consequently provided a physical rationale for examining DAPI, FITC, Cy3, and Cy5 separately rather than a dedicated supervisory mechanism within the GAN.

The aim of this work is to present and evaluate an improved VS pipeline that reuses clinically archived H&E slides to construct same-section fluorescence–H&E supervision and then transfers the learned representation to native AF inputs lacking paired H&E targets. Eosin-driven fluorescence and hematoxylin-associated attenuation are treated as channel-dependent optical contrast present in the source-domain fluorescence images, not as explicit neural-network constraints. The workflow combines image registration and tiling, optional SSCOR preprocessing, Pix2Pix Dense U-Net training and fine-tuning, and an evaluated CycleGAN normalization step that was subsequently discarded. Whole-slide virtual H&E outputs from canine and feline soft-tissue sarcomas (STS) and mast cell tumors (MCT) were assessed in SlideViewer v2.8 (3DHISTECH Ltd., Budapest, Hungary) using descriptive image metrics (FID and SSIM) together with semiquantitative evaluation by an experienced veterinary pathologist. This design separates the registered source-domain supervision used for model development from the native AF target domain used for inference.

## 2. Related Work

Deep learning (DL) has enabled VS of label-free tissue images, particularly using paired image-to-image translation. Pix2Pix [1] is a widely used framework for supervised image-to-image mapping and has been applied to AF-driven H&E stain generation [4,15]. The same workflow can also incorporate the SSCOR preprocessing method [7]. AF imaging leverages intrinsic tissue fluorophores, enabling VS without additional use of chemical dyes. Rivenson et al. [4] demonstrated paired AF-to-H&E virtual staining with preservation of histologically relevant structures. Using spatially registered AF↔H&E pairs, that study reported virtual images that retained histopathologically relevant information and showed strong agreement in pathologist-based evaluation [4]. The impact of multi-channel AF input contributing to final stain quality was explored by Loo [15]. As a result, the virtual H&E stains reproduced key morphologic features at cell levels (cytoplasm appearance, mitoses, nuclear lobation, nucleoli) and reproduced diagnostic features relevant for tumor subtyping, or differentiation between tumor, nontumor, and immune cells [15]. In our proposed transfer learning approach we also investigated if separating channels (DAPI, FITC, Cy3, Cy5) can reveal modality-specific features, which motivated our channel-wise analysis to assess how each spectral band contributes to the AF→H&E translation.

When producing paired samples is not feasible, unpaired translators such as CycleGAN [2] and CUT [3] have been adopted for virtual histopathology. Applications include ultraviolet photoacoustic microscopy [8]; the broader preprocessing pipeline can incorporate SSCOR-based stripe correction [7]. Other examples include photoacoustic-to-H&E translation without pixel alignment [18], multiplexed profiling [12], and stain adaptation for invasive carcinoma classification [11]. Recent work explored dual contrastive learning objectives for improving virtual H&E stain quality when applied to brightfield microscopy patches of skin tissue [14].

Stain conversion across modalities has also been explored using the hyperspectral measurements in transmittance mode. Biswas [22] parallels our idea to reuse H&E-stained slides: the authors proposed a CycleGAN-based approach to generate Verhoeff’s van Gieson stain from hyperspectral H&E transmittance images, combining unpaired adversarial training with subsequent supervised refinement after SURF-based pixel alignment to obtain paired ground truth. The implementation in Biswas [22] provides a relevant precedent for combining unpaired translation with registration-derived supervised refinement. However, in contrast to Biswas [22], our workflow uses registered same-section fluorescence–H&E pairs for source-domain supervision and channel-specific fine-tuning, while native label-free AF from adjacent unstained sections is introduced only during inference.

For supervised paired training, high-resolution whole-slide data require careful spatial registration because the fluorescence and H&E patches must correspond to the same tissue region. ORB features [25] with binary descriptors [26], followed by RANSAC-based geometric outlier rejection [27], [28], remain a robust choice for aligning eosin-enhanced fluorescence to H&E. Preprocessing via HSV masking [29] helps isolate tissue regions before alignment. OpenSlide v.4.0.0 [30] and DeepZoom tiling pipelines enable scalable processing of gigapixel slides, a necessity for training GANs on 512 × 512 patches.

The following preprocessing stages can support both paired and unpaired translation workflows. Scanner-induced stripe artifacts degrade fluorescent image mosaics. The SSCOR approach [7] explicitly models stripe patterns and trains a correction network to remove them while preserving tissue morphology; this method builds on broader efforts to mitigate striping in fluorescence imaging [31]. After VS, color variability remains an issue. Traditional stain normalization via color deconvolution (e.g., Macenko’s method [32]) has largely given way to DL-based chromatic harmonization. CycleGAN-style normalization can learn a target H&E color distribution without paired references, complementing Pix2Pix outputs.

Quantitative assessment commonly employs the Fréchet Inception Distance (FID) to compare generated and real feature distributions [33], alongside the Structural Similarity Index (SSIM) for structural fidelity [7]. FID and SSIM are descriptive image-quality metrics and do not, by themselves, establish diagnostic correctness; semiquantitative scoring frameworks—like the one we adopt—allow pathologists to grade staining uniformity, image clarity, and tissue accuracy, providing clinically meaningful validation alongside FID/SSIM analyses.

## 3. Materials and Methods

### 3.1. Tumor Sample Preparation for Histochemical H&E Staining and Slide Imaging

Formalin-fixed, paraffin-embedded (FFPE) tissue blocks containing tumor samples were obtained from routine biopsy submissions to a commercial veterinary pathology laboratory (Matise Veterinary Pathology Service, Latvia). Data collection and the use of animal-derived material in this study were approved by the Ethical Committee of Animal Welfare and Protection, Latvia University of Life Sciences and Technologies, Jelgava, Latvia, authorization No. DZLAEP-2021/3. Samples originated from client-owned dogs and cats for which excisional surgery had been recommended by the attending veterinarian. For this study, representative blocks from 13 STS and 12 MCT were selected. Tissues had been fixed in 10% neutral buffered formalin, trimmed, processed routinely through graded alcohols and xylene, and embedded in paraffin. From each FFPE block, two serial 5 µm sections were cut and deparaffinized. One section was left unstained (without coverslip) and scanned using a Pannoramic 250 Flash III slide scanner (3DHISTECH Ltd., Budapest, Hungary) equipped with a 20x/0.8 NA Plan-Apochromat (Carl Zeiss AG, Jena, Germany) objective and Lumencor SOLA light engine (Lumencor, Inc., Beaverton, OR, USA). The next serial section from each block was stained with H&E and coverslipped according to standard histochemical protocols.

Both unstained and H&E-stained slides were scanned using DAPI (EX 377/50 nm, EM 447/60 nm), FITC (EX 485/20 nm, EM 524/24 nm), Cy3 (EX 531/40 nm, EM 593/40 nm), and Cy5 (EX 628/40 nm, EM 692/40 nm) filter channels, using a single point raster scan time of 200–500 ms (DAPI, FITC and Cy3) or 1000 ms (Cy5). All histochemically stained and virtually stained whole-slide images (WSIs) were viewed and analyzed using 3DHISTECH SlideViewer (3DHISTECH Ltd., Budapest, Hungary) v2.8 (Build 2.8.0.216379) by a single board-certified veterinary pathologist (I.M.-V.H.).

### 3.2. Data Preparation, Preprocessing, and Channels

One of the most critical steps for successful NN training and the subsequent VS procedure is dataset preparation. Because different models and training techniques were used in this research, the data collection process was adapted to each proposed task. For example, for Pix2Pix training, multispectral fluorescence WSIs acquired from the H&E-stained sections were registered to brightfield H&E WSIs acquired from the same physical sections. The registration pipeline was adapted from our prior work [34].

**Figure 2:**
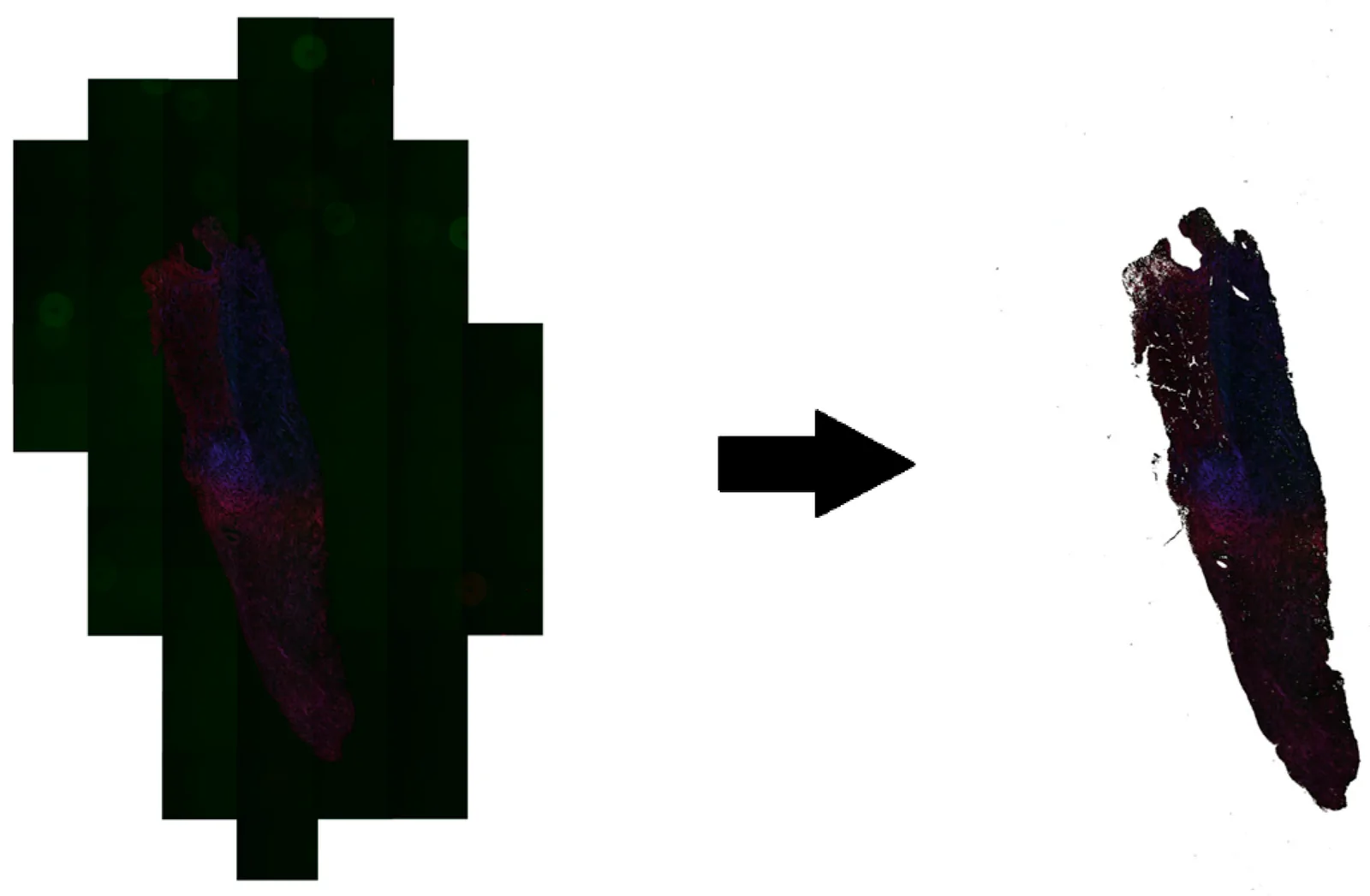
Fluorescence image of an H&E-stained section (**left**) and the corresponding HSV tissue mask used before registration (**right**).

A vital step in the registration process is Hue Saturation Value (HSV) filter masking [29]. The HSV filter mask isolates parts of an image (background in the fluorescence image of the H&E-stained section) shown in Figure 2 by keeping only the pixels whose hue, saturation and value fall within a chosen color range. Without this step, the registration algorithm would not reliably identify corresponding regions between the image pairs and therefore could not establish reliable registration.

**Figure 3:**
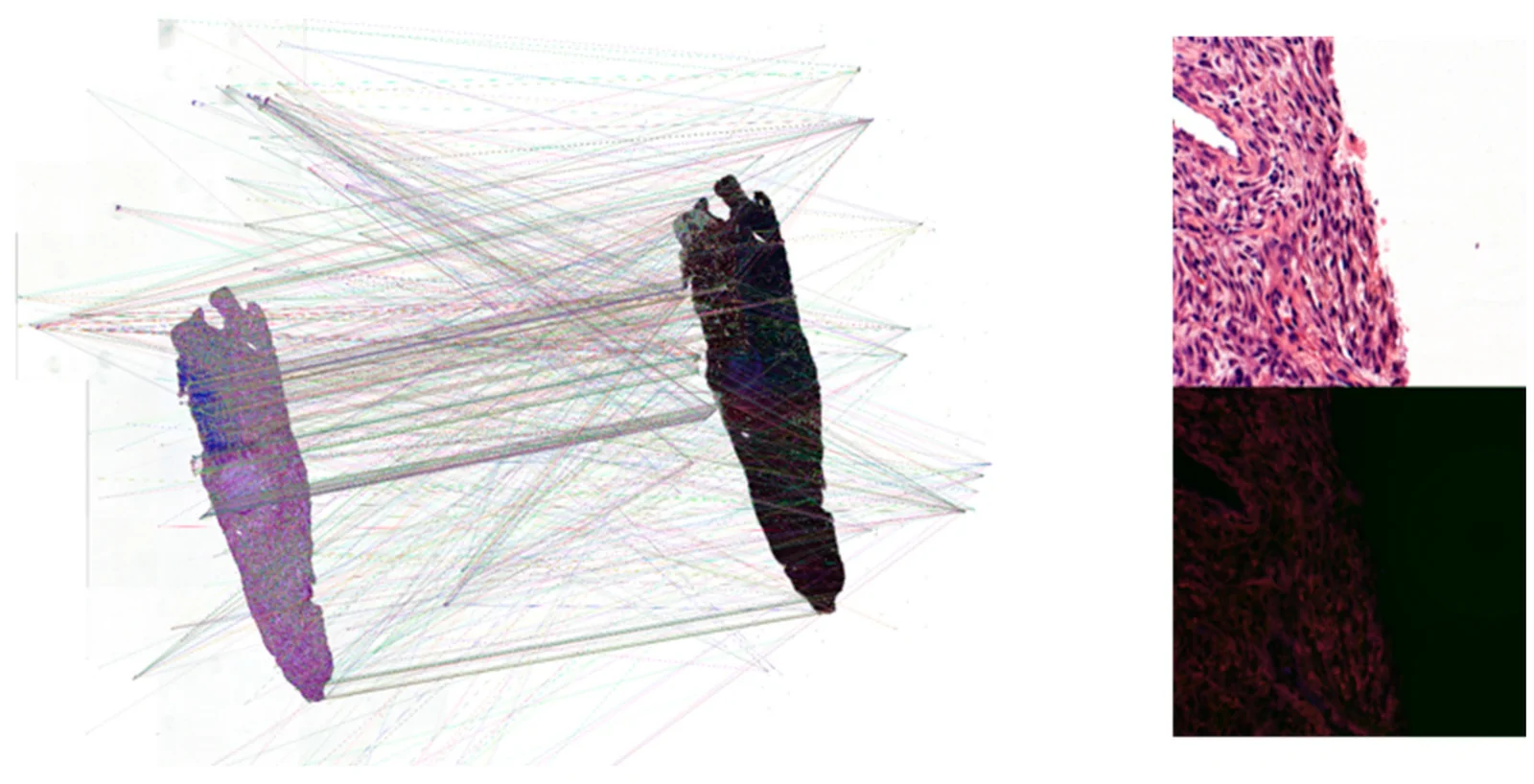
ORB feature matching used for image registration (**left**) and a representative registered fluorescence–H&E tile pair (**right**).

ORB-based registration first detects distinctive ORB keypoints in each image and computes their binary descriptors [25]. These descriptors are then matched between images using a brute-force approach with Hamming distance [26], after which unreliable correspondences are filtered out. To robustly estimate the geometric relationship between the images, the remaining matches are processed using the RAndom SAmple Consensus (RANSAC) algorithm [27,28], which iteratively selects minimal subsets of correspondences to estimate a homography while discarding outliers that do not conform to a consistent geometric model. The final result is a 3 × 3 homography matrix that represents the projective transformation between the two image planes and is used to warp and spatially align one image to the other, as illustrated in Figure 3.

In total, 60 WSIs were successfully registered for the model training procedure. Furthermore, several challenges were overcome compared to the previous iteration of the VS. As the WSI dimensions are determined in thousands of pixels, the magnification factor plays a crucial role when images are tiled and prepared. WSIs were subdivided into fixed-size 512 × 512 pixel tiles to enable efficient downstream processing and learning. Each slide was first loaded at a predefined resolution level and, if required, uniformly resized to control memory usage. The OpenSlide [30] v4.0.0 was used to access the WSI as a virtual multi-resolution slide, and OpenSlide’s DeepZoomGenerator was applied to generate a grid of non-overlapping tiles across the entire image extent [30]. The deepest available tiling level was selected to ensure maximal spatial coverage at the chosen resolution. All tiles were extracted without overlap and saved individually using a consistent coordinate-based naming scheme within slide-specific directories, allowing scalable storage and straightforward indexing for subsequent analysis.

A modified Stripe Self-CORrection (SSCOR) GAN model [7] was employed to reduce stitching artifacts in AF WSIs by effectively suppressing stripe-like artifacts and other scanner-induced nonuniformities commonly present in WSIs. In AF whole-slide imaging, stripe artifacts arise because the slide is scanned sequentially in narrow strips, and small variations in illumination, detector gain, or exposure between scan lines are not perfectly normalized during stitching [31]. These acquisition inconsistencies are amplified by the low signal-to-noise ratio of AF, producing stripe patterns unrelated to tissue structure.

**Figure 4:**
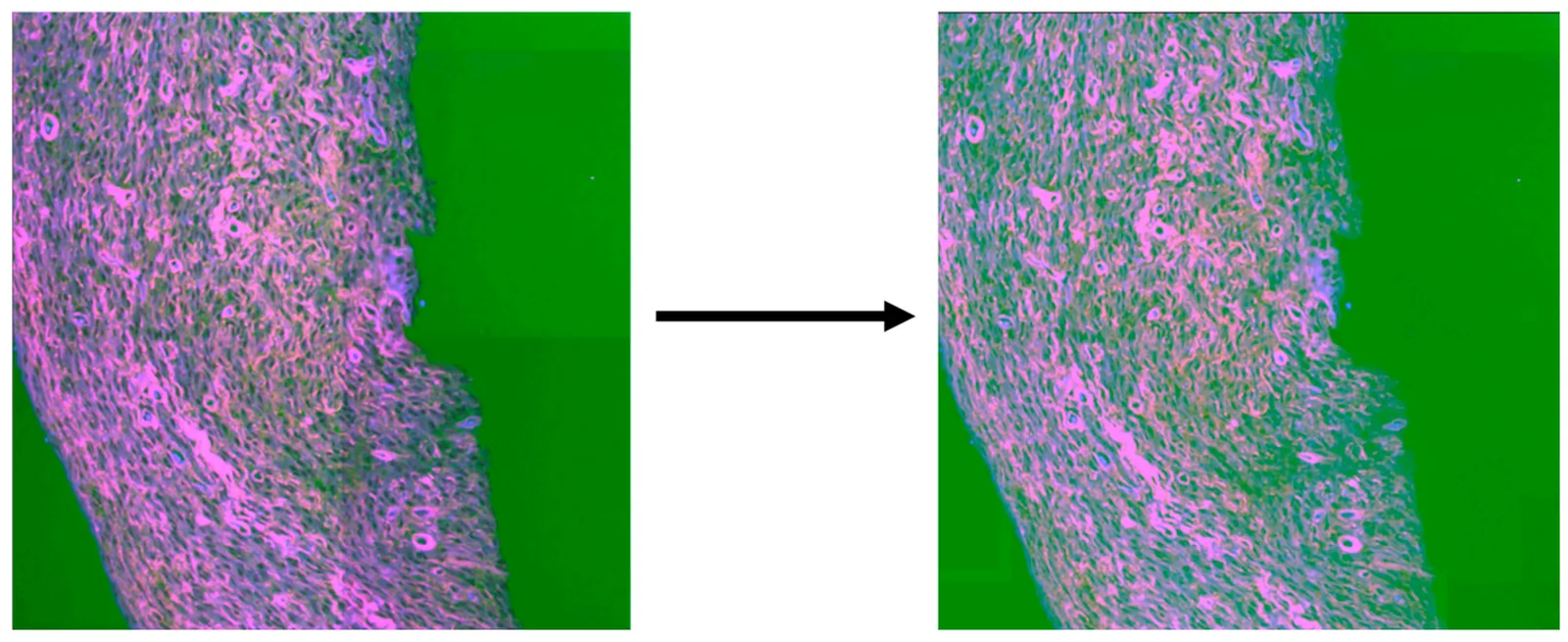
Autofluorescence stripe artifact correction before and after SSCOR processing.

The modified SSCOR network was applied as a preprocessing experiment to suppress stripe-like acquisition artifacts while preserving tissue morphology and intensity relationships. Importantly, SSCOR-processed images were not used to train or fine-tune Pix2Pix(v2); the trained staining model was kept fixed and evaluated on raw and SSCOR-processed inputs. SSCOR therefore functioned as an input-domain transformation rather than as a training regularizer. Its effect was assessed empirically because altering AF intensity and texture can also introduce a domain shift relative to the data used to train Pix2Pix(v2), as shown in Figure 4.

**Figure 5:**
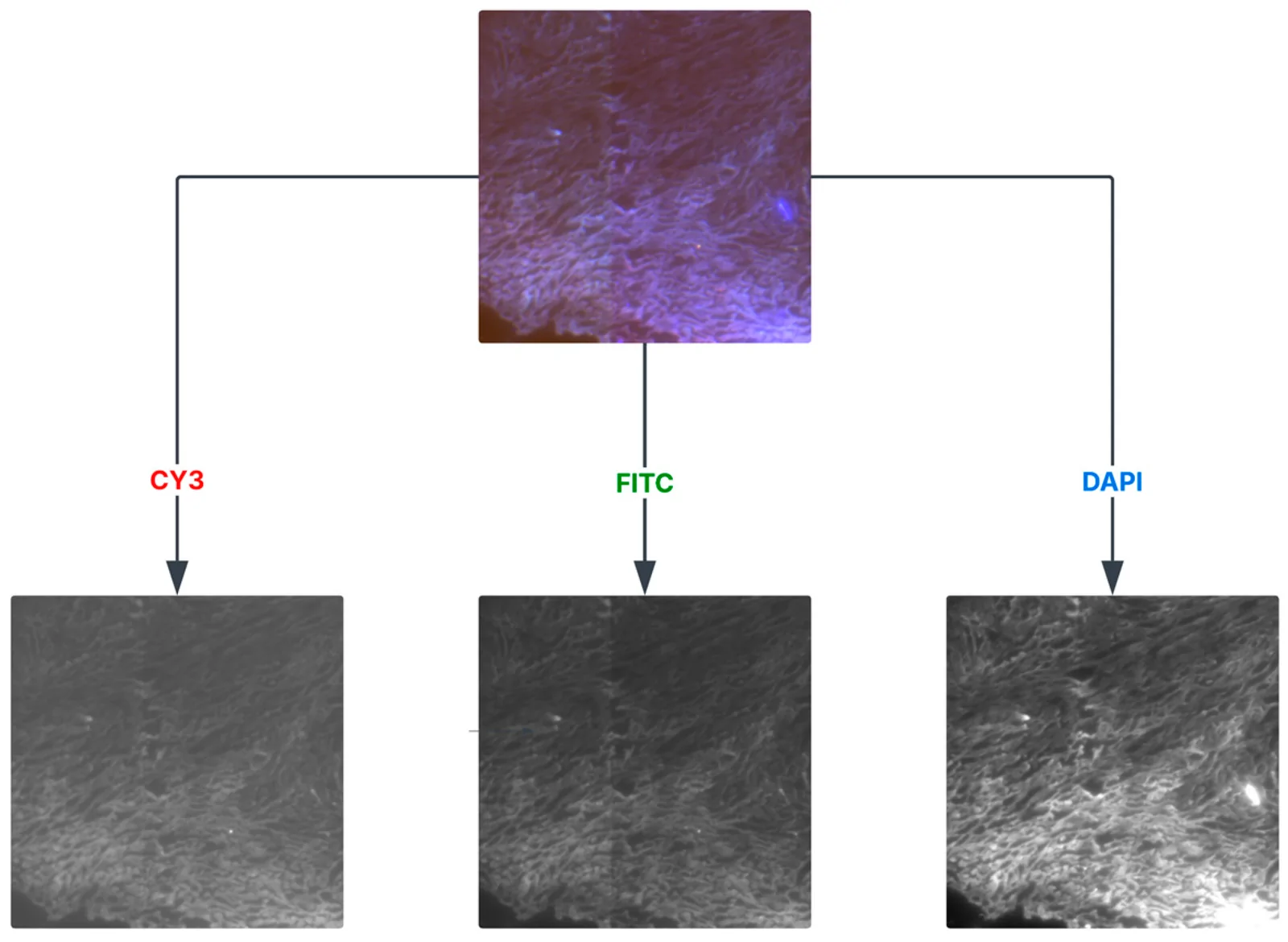
Channel decomposition of autofluorescence image into Cy3, FITC, and DAPI.

After registration, fluorescence scans of the H&E-stained sections were decomposed into individual spectral channels to construct the channel-wise paired training data (Figure 5). The original .mrxs WSI was converted to .tiff using SlideMaster v2.6 (Build 2.6.0.196850 RTM) (3DHISTECH Ltd., Budapest, Hungary), and the extractable channels were converted to single-channel grayscale WSIs in Fiji (ImageJ v1.54p). The same DeepZoom tiling procedure was then used to generate 512 × 512-pixel patches. RGB/color-encoded versions of the extracted channels were also retained for the representation experiments. Native AF WSIs from the adjacent unstained sections were processed separately in the same channel-wise manner for target-domain inference; they were not used as paired H&E targets.

After inference, generated 512 × 512-pixel tiles were stitched at their original coordinates to reconstruct the virtual-stained WSI, converted to .mrxs format, and reviewed in SlideViewer by the pathologist. In total, 14 datasets comprising more than 272 WSIs were created and stored on a local server for model development, inference, and evaluation.

### 3.3. Pix2Pix Improvement

In our initial implementation of the Pix2Pix Dense U-Net model [34], the main limitation was the degradation of image quality when zooming into regions containing cellular structures. This effect can be attributed to the properties of the training data: the model was trained on image fragments captured with a 20× objective, which were resampled into 512 × 512-pixel PNG patches. Because PNG is lossless, the observed loss of fine detail is attributable to the effective spatial resolution and resizing/extraction pipeline rather than image compression. Consequently, the earlier patches contained insufficient fine-scale morphological information for reliable high-magnification reconstruction.

**Figure 6:**
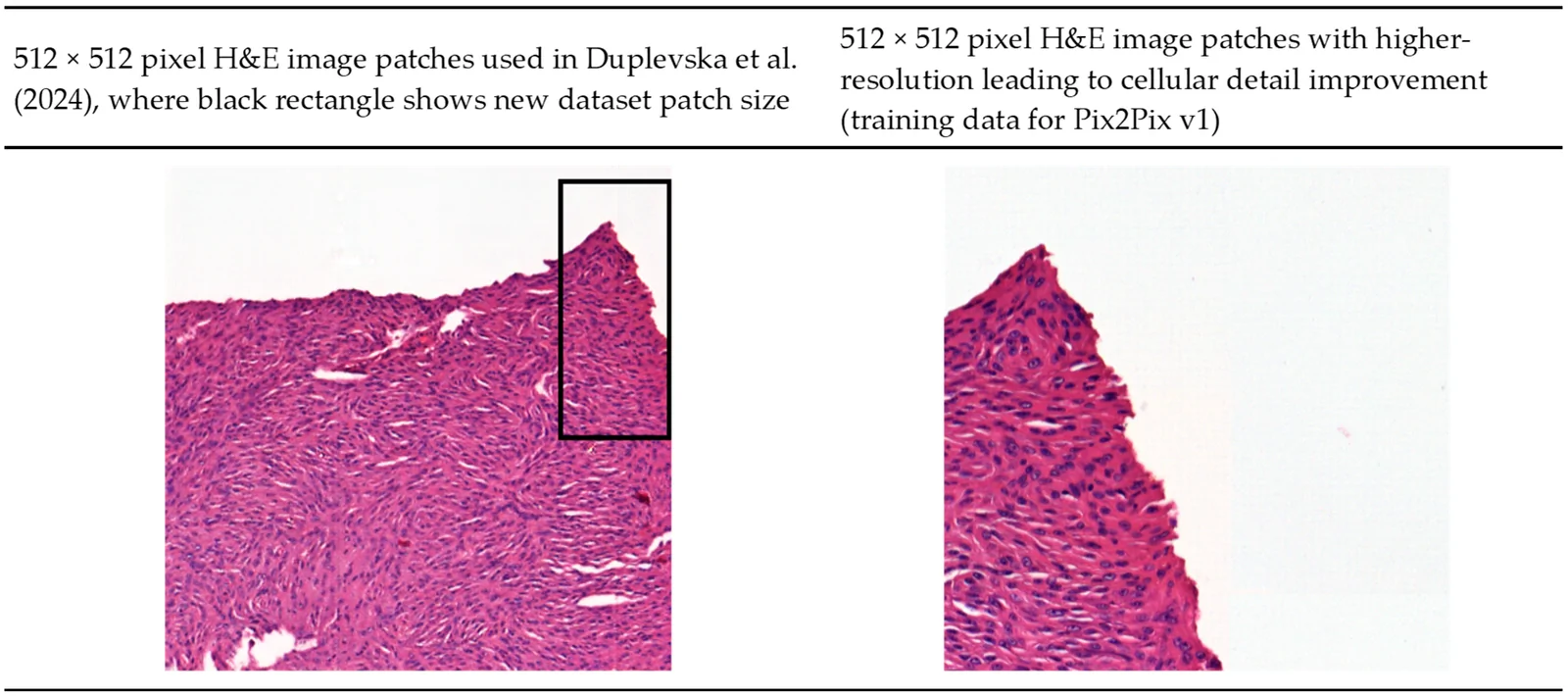
Comparison of representative images from initial dataset [34] and higher-resolution dataset developed in the present study (it also corresponds to column 1 in Supplementary Figure S1). The original output image dimensions were similar in (**left**) and (**right**) panels; however, for visualization purposes the (**left**) panel image has been upscaled to match the tissue regions of interest (ROIs) to illustrate the resolution improvement. Database case #1160he: fibrosarcoma, grade II, located in the skin between the shoulders of an 8-year-old mixed breed cat.

Since high resolution TIFF files could not be processed directly due to computational constraints, the magnification level of the training dataset was adjusted. This modification increased the amount of fine-scale structural information available in each training patch and therefore provided a stronger input signal for learning local texture and morphology. Figure 6 presents a comparative analysis of images from the previously published dataset [34] and from the newly registered dataset used in this study. Identical cellular regions were selected, allowing a clear assessment of the improvement in visualization quality.

When the previously trained Pix2Pix(v1) model was applied to the higher-resolution paired dataset without additional optimization, it reproduced the overall H&E color distribution in some regions but also produced pixelation, striping, noise, and incomplete or blank areas (Supplementary Figure S1, column 1). These failures indicated a mismatch between the earlier training distribution and the revised higher-resolution data.

**Figure 7:**
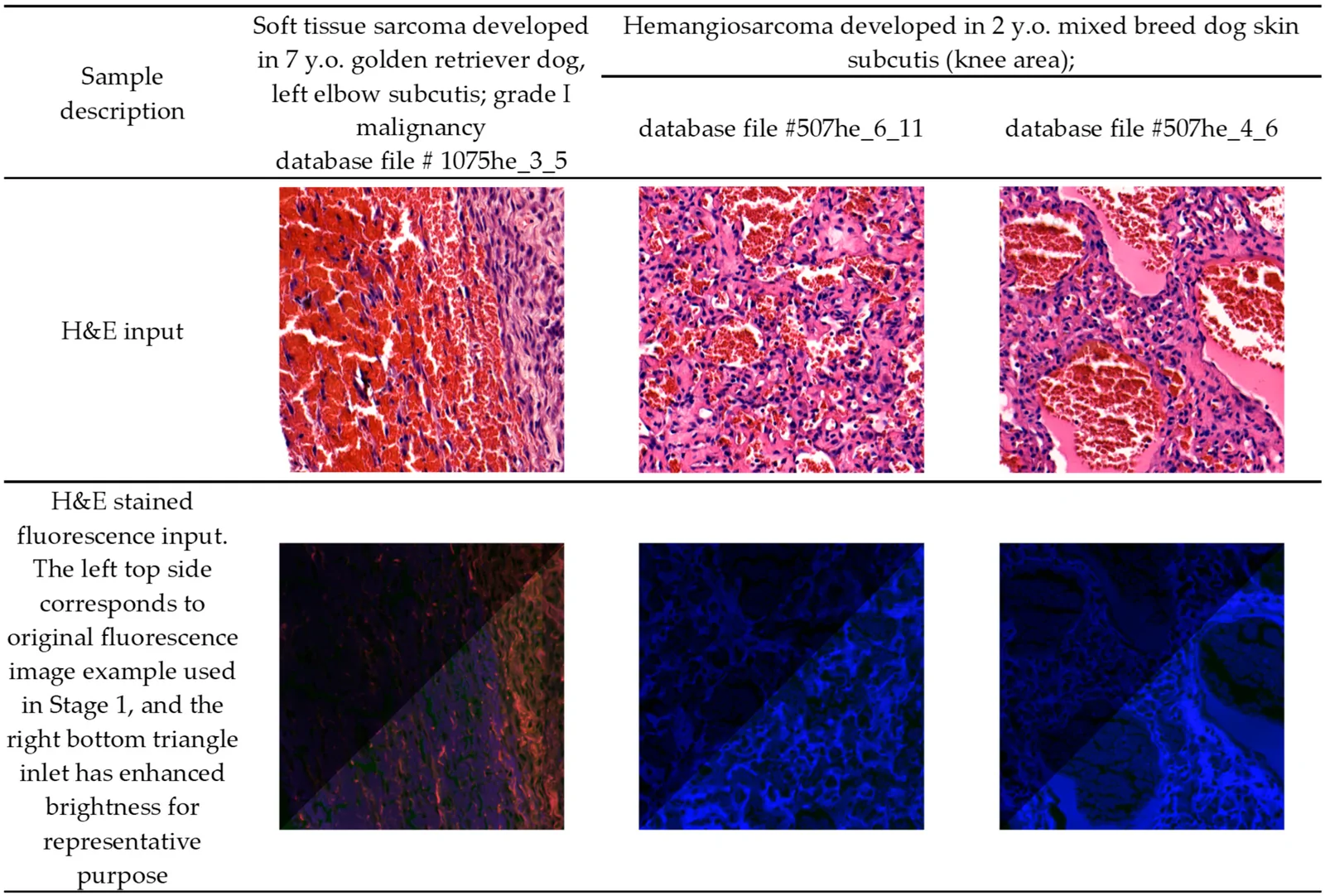
Representative examples of images comprising fine-tuning dataset (training data for Pix2Pix (v2)).

Pix2Pix(v2) was obtained by fine-tuning Pix2Pix(v1) on 300 manually selected, high-quality registered fluorescence–H&E patch pairs drawn from 1444 candidate pairs in the expanded dataset (Figure 7). Each pair consisted of a fluorescence patch from an H&E-stained section and the spatially corresponding brightfield H&E patch from the same physical section. During fine-tuning, random center-crop augmentation used a crop factor between 0.2 and 0.5, followed by resizing to 512 × 512 pixels. Patches were selected according to the following criteria:

- Absence of technical defects—images with intensity saturation or defocus were excluded.
- Minimal background regions—white or black background areas were allowed to cover no more than 10% of the total image area. Training patches of 512 × 512 pixels were selected such that cellular structures were clearly visible across the entire patch, with minimal background present only near the center as a dividing region.
- Morphological diversity—patches containing multiple tissue or cellular types were preferred, while homogeneous samples were excluded.

The Pix2Pix(v1) and Pix2Pix(v2) models used the same modified Dense U-Net generator and conditional PatchGAN discriminator. Training used 512 × 512-pixel patches and a batch size of 4. Adam optimizers used learning rates of 2 × 10−4 for the generator and 1 × 10−4 for the discriminator (β1 = 0.5). The generator objective combined adversarial binary cross-entropy with an L1 reconstruction term (λL1 = 100). Binary-cross-entropy label smoothing of 0.3 was used in the adversarial training implementation. Checkpoints were saved every 5000 optimization steps. Training was stopped manually after the loss curves reached a stable plateau, and checkpoint 46 was retained as Pix2Pix(v2). All Pix2Pix experiments were implemented in Python 3.8 (Python Software Foundation, Wilmington, DE, USA) with TensorFlow v2.4 and executed on an NVIDIA A100 GPU with 40 GB VRAM (NVIDIA Corporation, Santa Clara, CA, USA).

Visual comparison after fine-tuning showed fewer blank or ignored regions and more complete H&E-like rendering across several slides. These observations describe image-level output completeness rather than proof of improved cellular fidelity, which was evaluated separately using quantitative metrics and pathologist scoring.

**Figure 8:**
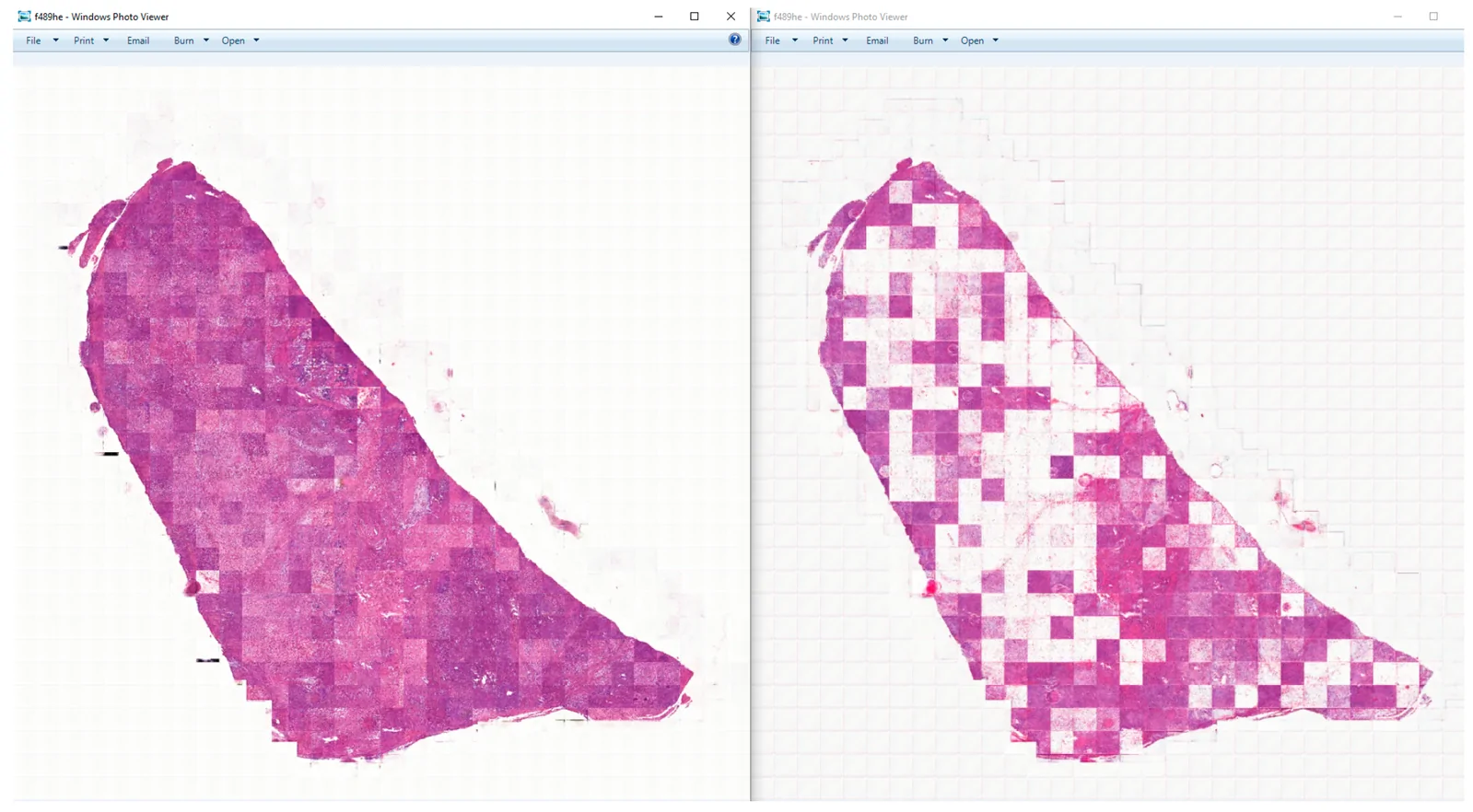
Comparison of fine-tuned virtual staining with the initial virtually stained (VS) approach. Representative image of grade II soft tissue sarcoma, identified in the elbow skin of 10-year-old Jack-Russel terrier (database file #489he). (**Left panel**) result of eosin fluorescence enhanced image translation to VS H&E by applying Pix2Pix(v2). (**Right panel**) result of eosin fluorescence enhanced image translation to VS H&E by applying Pix2Pix(v1).

Figure 8 presents an example where visual improvements in VS are clearly observable. It provides a comparative illustration: the left image shows the result obtained using the fine-tuned algorithm, while the right image displays the output generated by the original Pix2Pix Dense model.

### 3.4. CycleGAN for Image Stain Normalization

CycleGAN was evaluated as an optional post-processing step to reduce color variability in the Pix2Pix-generated virtual H&E images. This approach is frequently employed in virtual H&E stain normalization [35,36] as it allows heterogeneous H&E samples to be transformed into a consistent chromatic style—for example, more blue, purple, red, or pink, depending on the most common appearance within the dataset. In principle, such normalization makes many morphological and structural cues more comparable across slides; for instance, the color of red blood cells becomes homogenized. To accomplish this, a CycleGAN model was trained for image-to-image style transfer.

In this setup, CycleGAN was used only as a post-generation stain-normalization model: it mapped heterogeneous virtual-H&E outputs (domain X) toward a uniform pink H&E reference style (domain Y) without requiring paired X–Y samples. The training set comprised 2000 512 × 512-pixel image patches: 1000 source-domain patches with mixed or uneven color appearance and 1000 target-domain patches with a consistent pink appearance. The implementation used two generators for X→Y and Y→X mappings and two discriminators. Optimization combined adversarial loss, cycle-consistency loss, and identity loss. The adversarial terms encouraged each generated domain to match the corresponding real-domain distribution, cycle consistency constrained round-trip reconstruction, and identity loss penalized unnecessary changes when an image was already presented to the generator of its own domain.

Adversarial loss:

*L*_GAN_(*G*, *D_Y_*, *X*, *Y*) = *E_y_*_∼*Y*_ (log(*D_Y_*(*y*))) + *E_x_*_∼*X*_ (log(1 − *D_Y_*(*G*(*x*)))) (1)

Cycle-consistency loss:

*L*_cyc_(*G*, *F*) = *E_x_*_∼*X*_ (‖*F*(*G*(*x*)) − *x*‖_1_) + *E_y_*_∼*Y*_ (‖*G*(*F*(*y*)) − *y*‖_1_) (2)

Identity loss:

*L*_id_(*G*, *F*) = *E_y_*_∼*Y*_ (‖*G*(*y*) − *y*‖_1_) + *E_x_*_∼*X*_ (‖*F*(*x*) − *x*‖_1_) (3)

Final objective:

*L* = *L*_GAN_(*G*, *D_Y_*, *X*, *Y*) + *L*_GAN_(*F*, *D_X_*, *Y*, *X*) + *λ*_cyc_ *L*_cyc_(*G*, *F*) + *λ*_id_ *L*_id_(*G*, *F*) (4)

In the implementation used here, *λ*_cyc_= 5, and the identity term was weighted as *λ*_id_ = 0.5 × *λ*_cyc_. Training used 512 × 512-pixel images for 400 epochs with a batch size of 23. Adam optimizers used a learning rate of 2 × 10^−5^ and *β*_1_ = 0.5 for both generators and discriminators.

In Equations (1)–(4), *x* ~*X* and *y* ~*Y* denote samples from the source and target domains; *E_x_*_∼*X*_ and *E_y_*_∼*Y*_ denote expectations over those domains; *G*: *X*→*Y* and *F*: *Y*→*X* are the two generators; *D_Y_* and *D_X_* are the corresponding discriminators; ‖ · ‖_1_ denotes the L1 norm; *λ*_cyc_ and *λ*_id_ weight the cycle-consistency and identity losses; and *L*_GAN_, *L*_cyc_, and *L*_id_ denote the adversarial, cycle-consistency, and identity terms in the total objective *L*.

Unlike Pix2Pix, CycleGAN does not require paired source–target images; it constrains unpaired translation by combining adversarial objectives in both directions with cycle-consistency and identity terms.

After training, the CycleGAN model was applied to Pix2Pix-generated virtual H&E patches as an optional post-processing step intended to reduce color variability and move outputs toward the pink reference distribution. Although the normalized images were more chromatically uniform in some cases, subsequent FID, SSIM, and expert evaluation showed reduced overall performance; therefore, CycleGAN normalization was not retained in the final VS workflow. Examples are shown in Supplementary Figure S1.

**Figure 9:**
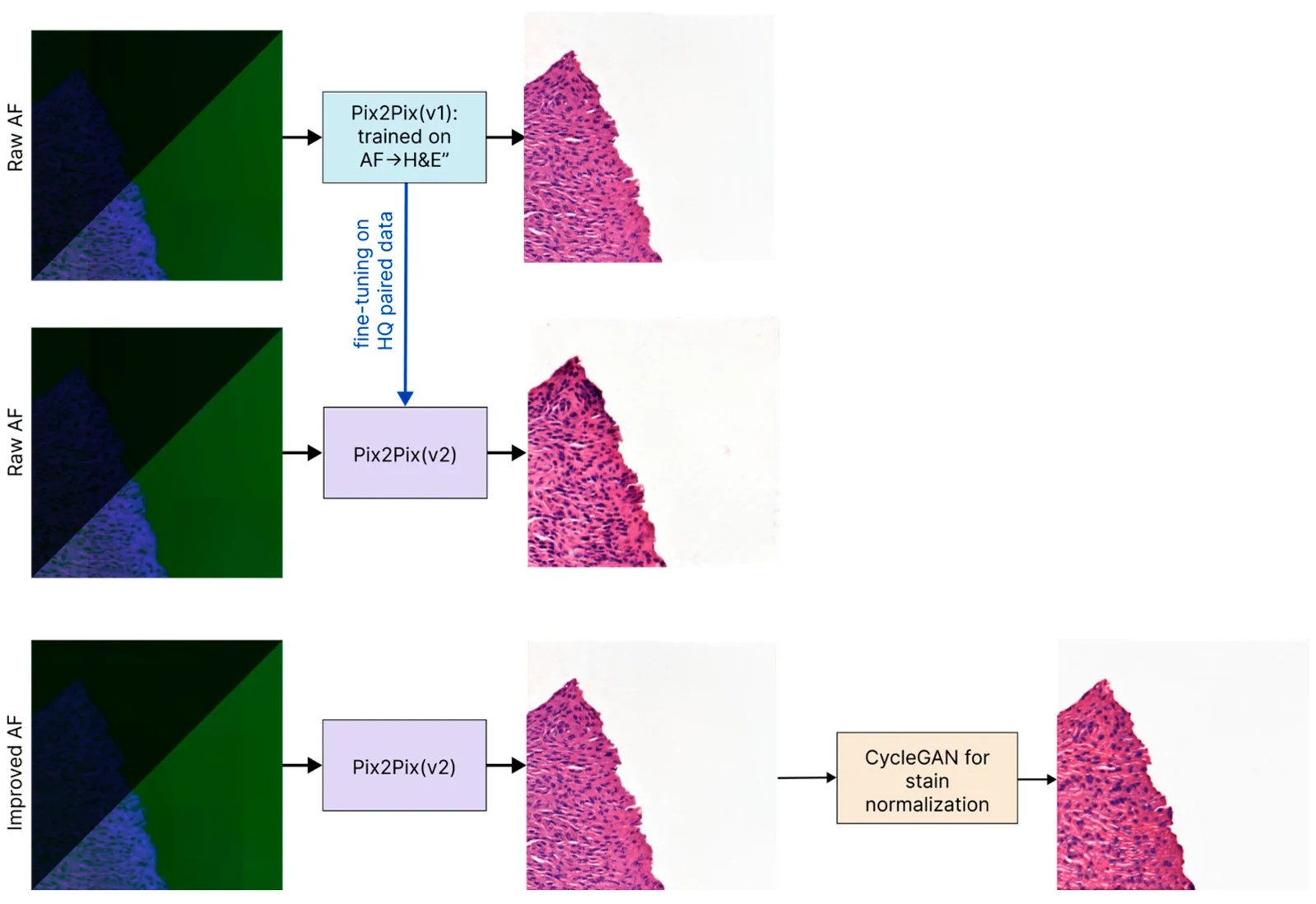
Evaluated virtual-staining workflow, including Pix2Pix(v1/v2), optional SSCOR input preprocessing, and optional CycleGAN output normalization.

Figure 9 summarizes the evaluated workflow. Pix2Pix(v1) was trained on the revised higher-resolution registered fluorescence–H&E dataset and fine-tuned on the zoom-augmented curated subset to obtain Pix2Pix(v2). SSCOR-processed inputs were not used during Pix2Pix(v2) training or fine-tuning. Instead, the fixed Pix2Pix(v2) model was applied to both raw and SSCOR-processed inputs to isolate the effect of preprocessing, and its outputs were evaluated with and without CycleGAN post-processing.

### 3.5. Pix2Pix for Separate Channel Processing

**Figure 10:**
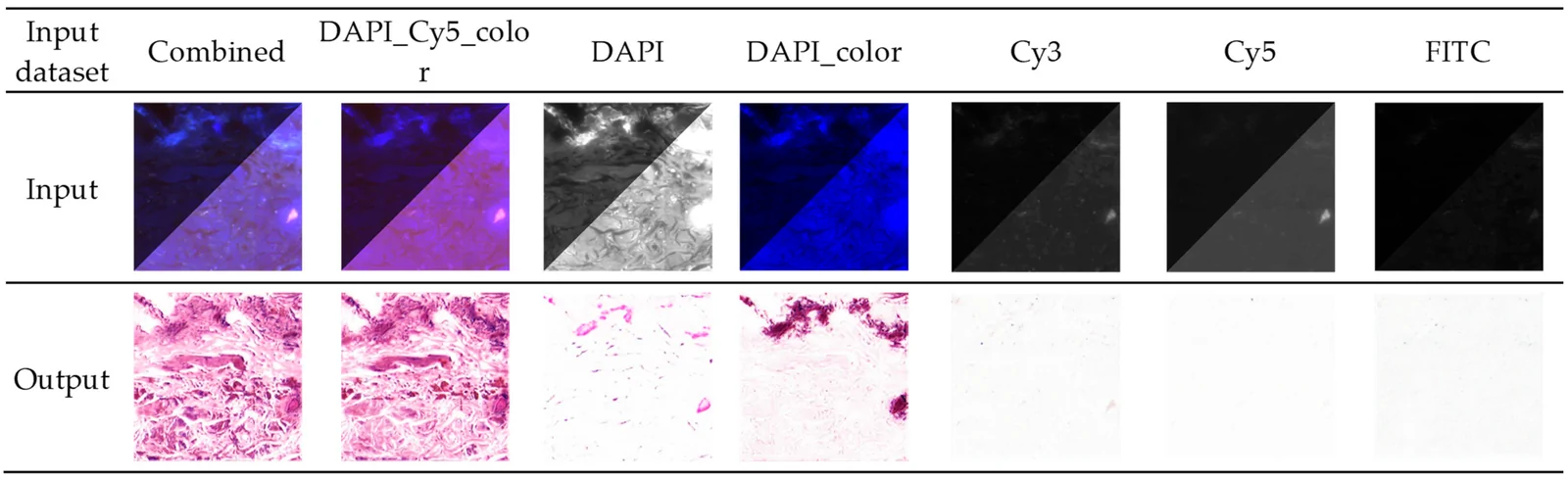
Direct Pix2Pix(v2) inference on isolated AF channel representations before channel-specific fine-tuning.

Stage 2 addressed the target-domain setting in which native AF images from unstained sections had no spatially matched H&E targets. As an initial test, Pix2Pix(v2) was applied directly to isolated DAPI, Cy3, Cy5, FITC, DAPI_color, and DAPI_Cy5_color representations from the unpaired AF dataset. The output depended strongly on channel and encoding, and several configurations produced incomplete or nearly empty virtual stains (Figure 10). This motivated channel-specific transfer learning rather than direct use of the Stage 1 model on the target-domain inputs.

**Figure 11:**
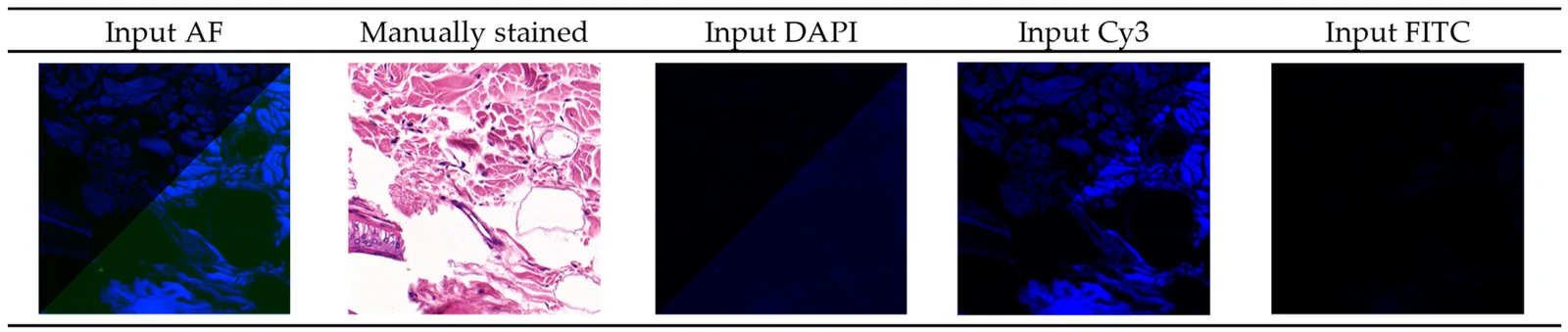
Example of channel-specific training data for Pix2Pix(v3). DAPI, Cy3, and FITC inputs were extracted from the fluorescence scan of the H&E-stained section and paired with the spatially corresponding brightfield H&E patch from the same tissue region. The native label-free AF image is shown for comparison with the target-domain input used at inference; it was not used as the H&E-paired training target. Four fluorescence channels were originally acquired (DAPI, FITC, Cy3, and Cy5), but vendor-limited RGB export during .mrxs-to-.tiff conversion in SlideMaster v2.6 (3DHISTECH Ltd., Budapest, Hungary) allowed only three channels to be extracted for channel-specific training, so Cy5 was excluded.

To construct the channel-specific fine-tuning data, the previously registered fluorescence–H&E source-domain pairs were decomposed into individual DAPI, Cy3, and FITC inputs. Each single-channel fluorescence patch remained spatially paired with the full brightfield H&E patch from the same tissue region. These data are therefore supervised channel-wise pairs; the H&E target is shared across channels rather than being channel-specific. The retained Pix2Pix(v2) checkpoint (checkpoint 46) was used to initialize each channel-specific Pix2Pix(v3) model before fine-tuning. Representative training inputs and targets are shown in Figure 11.

Separate Pix2Pix(v3) models were trained for RGB/color-encoded and grayscale representations. RGB inputs retained the three-channel tensor shape of Pix2Pix(v2), allowing all shape-compatible pretrained weights to be loaded directly. Grayscale inputs used a single input channel; because the first convolutional layer no longer matched the three-channel input shape, that layer was initialized for the one-channel configuration, while all remaining shape-compatible weights were loaded from Pix2Pix(v2). No layers were frozen during channel-specific fine-tuning.

**Figure 12:**
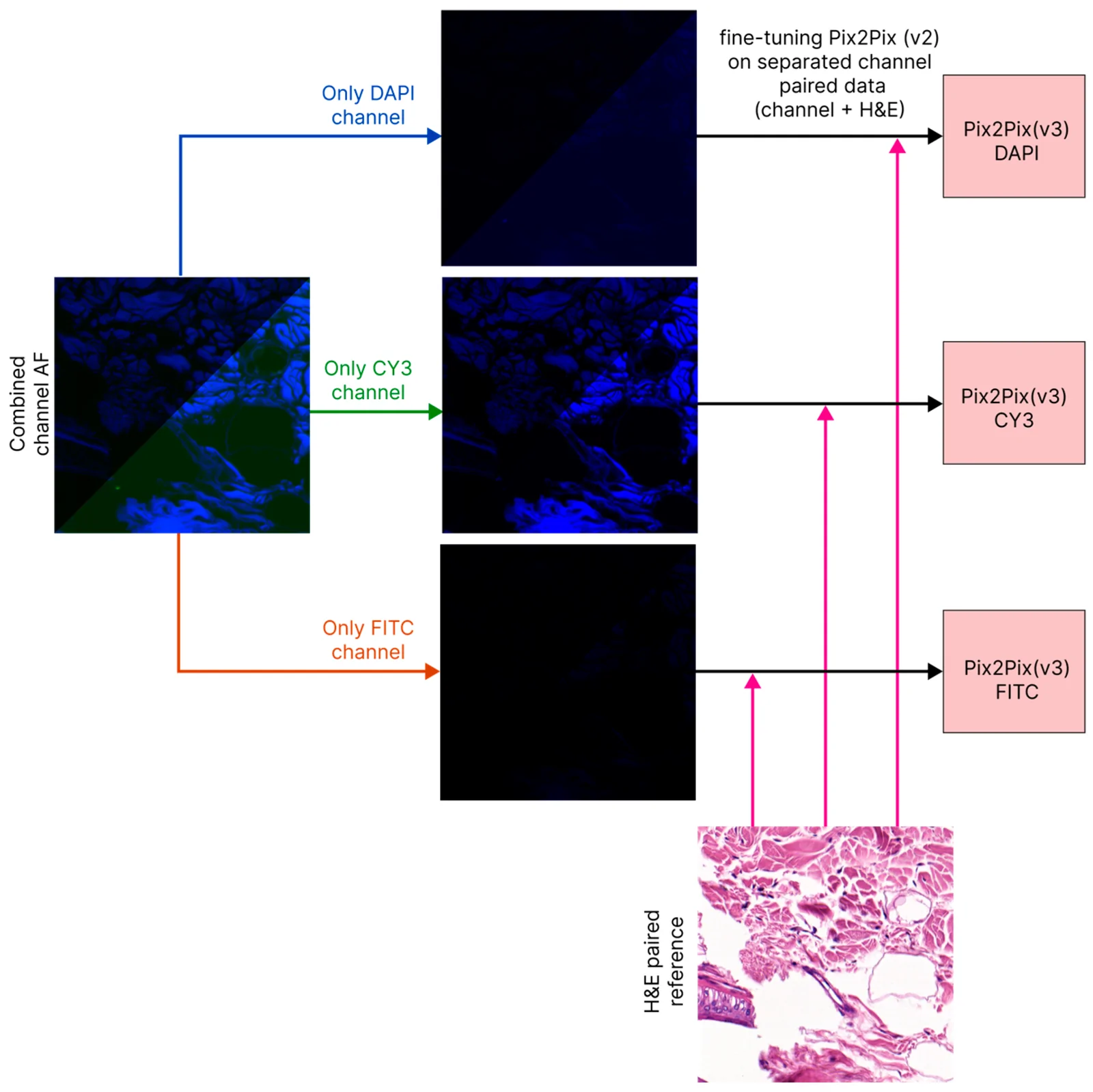
Channel-specific Pix2Pix(v3) transfer-learning scheme.

The Pix2Pix(v3) strategy is summarized in Figure 12. Starting from the same Pix2Pix(v2) checkpoint, independent DAPI, Cy3, and FITC models were fine-tuned on their corresponding registered single-channel fluorescence–H&E pairs. Stage 2 used a reduced learning rate of 2 × 10−5, and all trainable layers remained unfrozen. This design isolates the effect of channel representation while holding the pretrained initialization and training protocol constant. RGB and grayscale input representations were evaluated separately.

To isolate the contribution of pretrained initialization, matched channel-specific models were also trained from random initialization without loading Pix2Pix(v2). For each DAPI, Cy3, and FITC experiment, transfer-learning and from-scratch models used the same 1748 512 × 512-pixel patches, the same 80/20 patch-level train/test split (random seed 42), and the same held-out evaluation patches. RGB models used three input channels and grayscale models one. The from-scratch controls used the same Dense U-Net/PatchGAN architecture, batch size of 4, adversarial-plus-L1 objective (λL1 = 100), and Stage 2 optimizer settings as their transfer-learning counterparts. Because the split was performed at patch level rather than by WSI/case, this comparison estimates within-dataset patch-level performance and does not constitute independent slide-level validation.

The clinical cohort comprised 25 WSIs, each yielding approximately 100–300 512 × 512-pixel tiles depending on tissue area. For Pix2Pix(v3) fine-tuning and matched transfer-learning versus from-scratch evaluation, 1748 channel-specific image patches were retained after excluding completely non-informative or extremely low-content files. In contrast, whole-slide inference retained the natural heterogeneity of the tiled clinical images, including fully or partially empty regions and patches in which an individual fluorescence channel contained a weak or incomplete signal.

FID was calculated with torch-fidelity v0.4.0 [19] using the Inception-v3-compatible implementation and 2048-dimensional Inception features. Generated images and manually stained H&E reference images were supplied to torch-fidelity as saved image files. No additional manual resizing or normalization was applied specifically for FID calculation; inception-compatible preprocessing was handled internally by torch-fidelity. For the Pix2Pix(v3) transfer-learning and from-scratch comparison, identical AF evaluation patches and the same manually stained H&E reference set were used for each corresponding channel, so observed differences in FID under this single split reflect the training initialization and protocol rather than a change in the evaluation input or reference domain; they are not inferential estimates across independent retraining runs.

**Figure 13:**
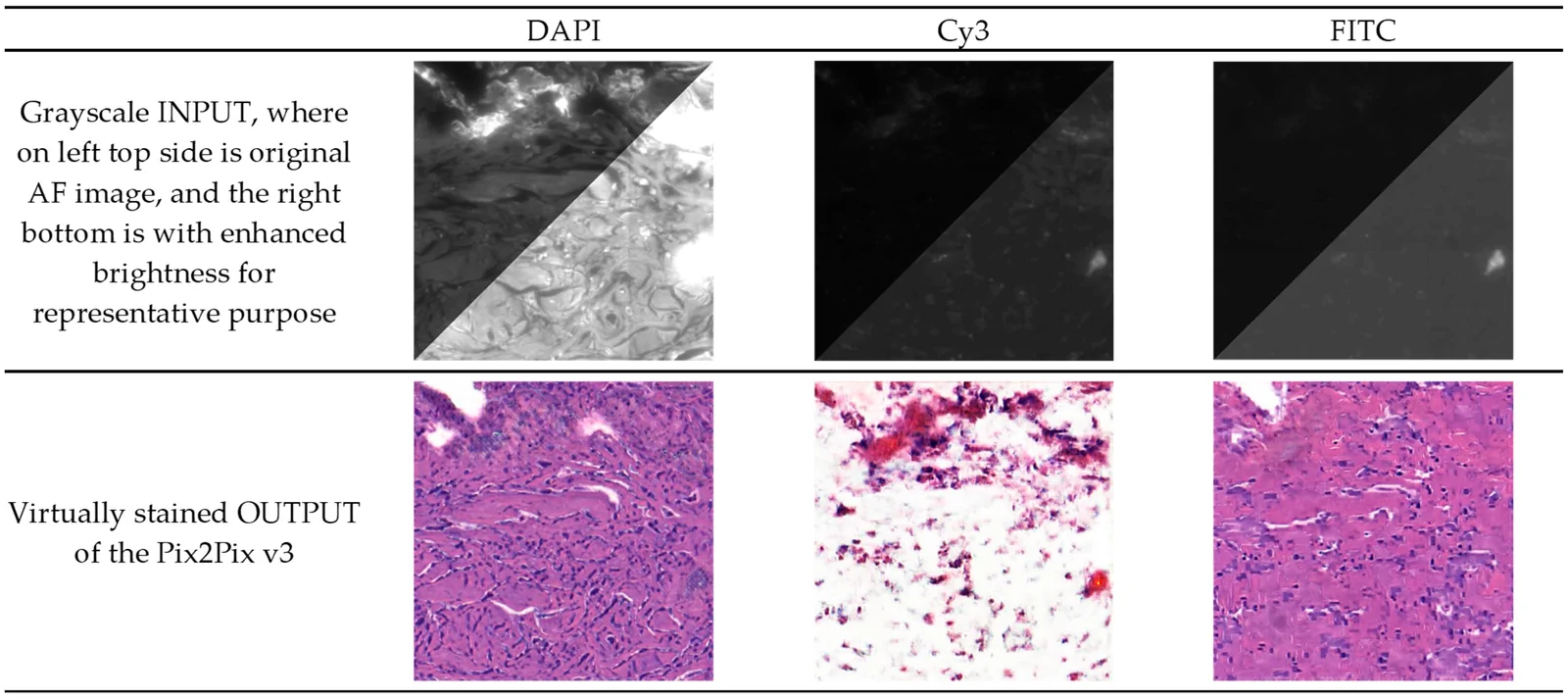
Representative Pix2Pix(v3) target-domain inference results for DAPI, Cy3, and FITC grayscale AF inputs.

After supervised channel-specific fine-tuning, Pix2Pix(v3) was applied to the target-domain native AF dataset, for which no paired H&E targets were available. The native AF WSIs were decomposed into individual channels and evaluated in RGB and grayscale representations. Relative to direct Pix2Pix(v2) inference on the same isolated channels, Pix2Pix(v3) produced more complete H&E-like outputs in the representative examples (Figure 13). Because target-domain H&E pairs were unavailable, these inference results were assessed at the whole-slide level using the pathology scoring framework rather than paired pixel-wise validation.

### 3.6. Scoring System for Assessment of Staining Quality, Image Quality and Tissue Accuracy in Virtually Stained Slides

A semiquantitative scoring framework was developed to assess the overall quality and accuracy of virtually stained whole-slide images relative to conventional H&E slides. All evaluations were performed by a single board-certified veterinary pathologist, who reviewed each WSI at variable magnifications using 3DHISTECH SlideViewer v2.8. The scoring exercise was designed as a single-observer semiquantitative assessment for method development and comparative evaluation; formal multi-observer validation of the scoring framework was outside the scope of the present study.

The scoring system was developed specifically for this study and was not derived from a previously validated histopathology scale (Table 1). Four categories were evaluated: (1) technical quality, (2) H&E staining, (3) image quality, and (4) accuracy. Each parameter was rated on an ordinal 0–3 scale, where 0 represented absent or severely unacceptable representation, 1 indicated poor representation, 2 moderate but still clearly suboptimal representation, and 3 optimal or only slightly suboptimal representation relative to a diagnostically acceptable conventional H&E slide. Technical quality (section-wide) comprised stain uniformity and tissue section completeness, each assessed over the entire tissue section. Technical quality, H&E staining, and image quality were scored based on the virtual WSI alone, whereas accuracy was scored by direct comparison with the matched ground-truth H&E slide (Table 1).

For the H&E staining, image quality, and accuracy categories, the pathologist reviewed each WSI at variable magnifications and identified, for each parameter, a subjectively best and worst region. Each parameter was scored separately in tumor tissue and in non-neoplastic tissue within each region, yielding four raw scores per parameter: tumor-best, tumor-worst, non-neoplastic-best, and non-neoplastic-worst. This design captured within-slide heterogeneity while preserving separate quality estimates for tumor and non-neoplastic compartments. For the accuracy category, non-neoplastic tissue components (epidermis, hair follicles/adnexa, smooth muscle, dermal collagen, subcutis, skeletal muscle) and tumor-related components (tumor cells, stroma, tumor margin) were assessed independently; components not present in a section were recorded as not applicable and excluded from denominators.

For each category, raw scores were converted to normalized sub-scores by dividing the sum of observed scores by the maximum possible score for the applicable items (range 0–1), with normalization performed separately for each tissue–region combination (tumor-best, tumor-worst, non-neoplastic-best, non-neoplastic-worst). For H&E staining, image quality, and accuracy, the category composite score was calculated as the sum of the four normalized sub-scores (range 0–4). Technical quality (whole-slide evaluation) was summarized as a single normalized category score (range 0–1). The slide total score was calculated as the sum of the technical quality score and the composite scores for H&E staining, image quality, and accuracy (maximum total score 13).

In addition to inclusion in the slide total score, tumor and normal composite sub-scores were retained separately (tumor-best + tumor-worst; non-neoplastic-best + non-neoplastic-worst) to enable downstream analyses comparing VS performance in neoplastic versus non-neoplastic compartments.

### 3.7. Pilot Comparison of AF Channel Input and Source Image Format

A four-case pilot comparison was performed to compare AF channel inputs and source image formats and to identify the highest-scoring configuration within this pilot dataset. Because configuration selection was based on only four cases and was not evaluated using an independent selection cohort or nested cross-validation, this comparison was considered exploratory. Four cases were selected (*n* = 2 STS; *n* = 2 MCT). For each case, virtual H&E WSIs were generated from four autofluorescence input configurations: DAPI only, FITC only, Cy3 only, and a three-channel composite (DAPI + FITC + Cy3). For each AF configuration, two source image formats were evaluated: RGB and grayscale. This 4 × 2 factorial design produced 8 VS configurations per case (total *n* = 32 virtual WSIs).

Virtual WSIs were scored using the predefined semiquantitative scoring framework (technical quality, H&E staining, image quality, and accuracy). The primary outcome was the total slide score, used to rank configurations and select the highest-scoring approach within the four-case pilot. A secondary outcome was the comparison of scores between tumor types (STS vs. MCT) and between tumor and non-neoplastic tissues to explore potential tumor-type-related differences in VS performance.

### 3.8. Evaluation of TL Virtual H&E in the Full Clinical Cohort 

Following the four-case pilot comparison of AF input and source image representation, the highest-scoring configuration (grayscale DAPI) was applied to the full study cohort comprising 13 STS and 12 MCT from dogs and cats. For each case, a virtually stained WSI was generated using the pilot-selected input settings and evaluated against the corresponding ground-truth H&E WSI.

Virtual WSIs were assessed using the predefined semiquantitative scoring framework, yielding category-level and total slide scores. Scores were generated for tumor and non-neoplastic tissue separately (best and worst regions), enabling compartment-specific analyses in addition to inclusion in the total slide score. Tumor-focused outcomes included the quality and accuracy of tumor cells, tumor stroma, and tumor margins, while non-neoplastic tissue outcomes reflected the same scoring parameters applied to normal tissue structures.

To complement ordinal scoring, the pathologist also recorded qualitative discrepancies between virtual and ground-truth H&E WSIs (e.g., recurrent artifacts or systematic differences affecting interpretability), documenting whether each discrepancy involved tumor tissue, non-neoplastic tissue, or both. Composite sub-scores and total slide scores (sums of ordinal component scores) were summarized as mean ± SD. Exploratory differences between STS and MCT were evaluated for each metric using two-sided Welch’s independent-samples t-tests (unequal variances). Because seven STS-versus-MCT comparisons were performed, *p*-values were adjusted for multiple testing using the Holm procedure. Both unadjusted and Holm-adjusted *p*-values were considered in interpretation. Given the small subgroup sizes (13 STS and 12 MCT) and the exploratory nature of these analyses, statistical comparisons were regarded as hypothesis-generating rather than confirmatory.

## 4. Results

### 4.1. Development and Refinement of the AF→H&E Virtual Staining Pipeline

**Figure 14:**
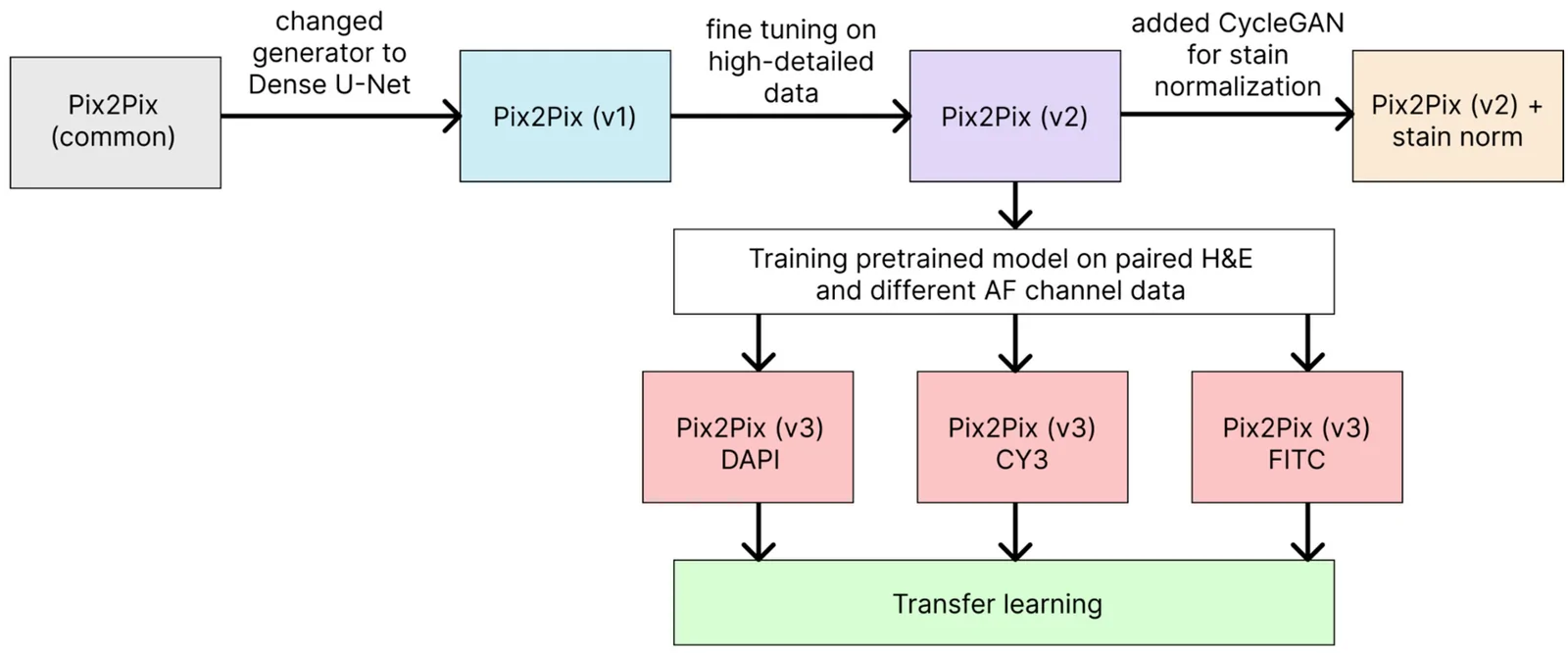
Transfer-learning virtual-staining workflow. Stage 1: same-section fluorescence–H&E registration → Pix2Pix(v1) training → Pix2Pix(v2) fine-tuning on 300 curated pairs selected from 1444 candidates. Stage 2: channel-wise DAPI/Cy3/FITC–H&E paired fine-tuning of Pix2Pix(v3) using 1748 patches → inference on native AF WSIs without paired H&E targets. The clinical cohort comprised 25 WSIs, each yielding approximately 100–300 tiles during whole-slide processing.

The model-development and transfer-learning workflow is summarized in Figure 14. Stage 1 comprises supervised training and fine-tuning on registered fluorescence–H&E pairs acquired from the same H&E-stained section. Stage 2 adapts the retained Pix2Pix(v2) weights to individual fluorescence channels using registered channel-wise pairs and then applies the resulting Pix2Pix(v3) models to native AF from unstained sections for which no paired H&E target is available.

Pix2Pix with a standard U-Net generator was used as the initial baseline. Because this configuration did not provide sufficiently stable H&E-like translation on the revised data, the generator was replaced with a Dense U-Net while retaining the conditional GAN formulation. The revised architecture improved output completeness and structural appearance at the tested patch scale, but limited effective input resolution still constrained high-magnification detail.

Pix2Pix(v1) was trained on the revised higher-resolution registered fluorescence–H&E dataset. The same model was then fine-tuned on the curated 300-pair, zoom-augmented subset to obtain Pix2Pix(v2). SSCOR-processed inputs were not used during either training step; Pix2Pix(v2) was therefore evaluated on SSCOR-processed inputs as a fixed model to measure the effect of preprocessing and domain shift.

CycleGAN was evaluated only as an optional post-processing normalization stage for Pix2Pix(v2) outputs. It was trained unpaired to map heterogeneous virtual-H&E color distributions toward a uniform pink reference domain. Although the transformation reduced color variability in some images, it increased FID, reduced SSIM, and received poorer expert scores under the tested configuration; it was therefore excluded from the final workflow.

Transfer learning was then evaluated for the native AF target domain. Direct application of Pix2Pix(v2) to isolated DAPI, Cy3, Cy5, and FITC channels from unstained AF often produced incomplete or nearly empty outputs. The retained Pix2Pix(v2) checkpoint was therefore used to initialize channel-specific Pix2Pix(v3) models.

Each Pix2Pix(v3) model was fine-tuned under supervised channel-wise pairing: a DAPI, Cy3, or FITC fluorescence patch from the registered H&E-stained source section was paired with the corresponding full brightfield H&E patch from the same tissue region. After this adaptation, the models were applied to native unpaired AF WSIs. Thus, the target-domain AF data were not used as paired supervision during optimization; transfer occurred through pretrained parameters followed by channel-specific supervised fine-tuning. The adapted models produced more complete virtual stains than direct Pix2Pix(v2) inference on isolated target-domain channels.

A detailed analysis of the virtually stained structures and their histological relevance, as assessed by the veterinary pathologist, is presented in the dedicated semiquantitative evaluation section.

### 4.2. FID for SSCOR-Improved and Raw AF Data

**Figure 15:**
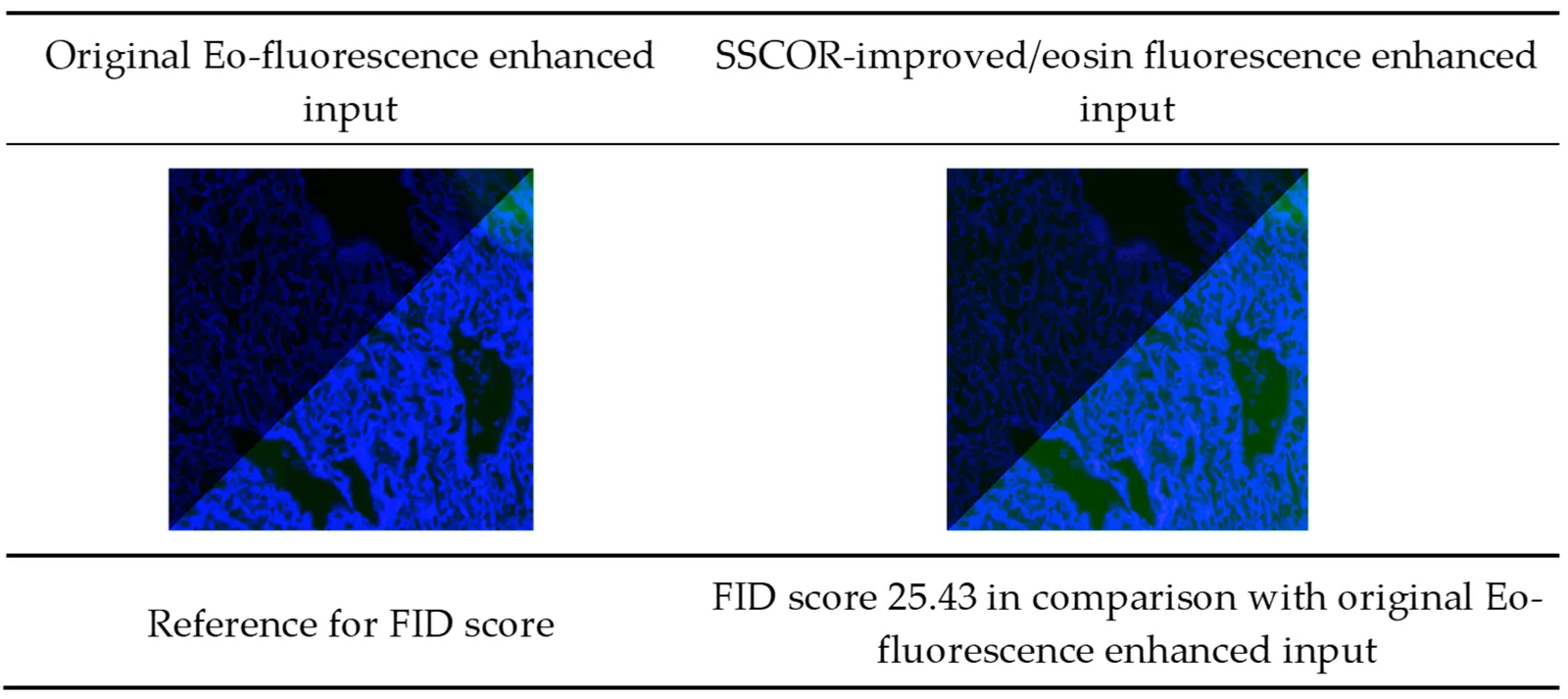
Distributional comparison of original and SSCOR-processed eosin-enhanced fluorescence inputs. The FID between the two input sets was 25.43.

SSCOR changes the input distribution seen by the fixed staining model. The FID of 25.43 between original and SSCOR-processed eosin-enhanced fluorescence inputs (Figure 15) quantifies a distributional difference between the two input sets; it does not by itself indicate that either input domain is superior. Because Pix2Pix(v2) was trained without SSCOR-processed inputs and was then applied unchanged to both domains, downstream differences in VS metrics can be interpreted as the effect of preprocessing and the associated domain shift rather than as a consequence of retraining.

### 4.3. FID Score for Pix2Pix Model

FID compares the multivariate distributions of Inception feature embeddings from generated and reference images; lower values indicate closer agreement between the two feature distributions. FID does not require one-to-one correspondence between generated and reference images, but its interpretation depends on comparable sample domains and a representative H&E reference set. In this study, FID was used as a descriptive distribution-level image metric rather than a measure of diagnostic accuracy or cellular fidelity.

**Figure 16:**
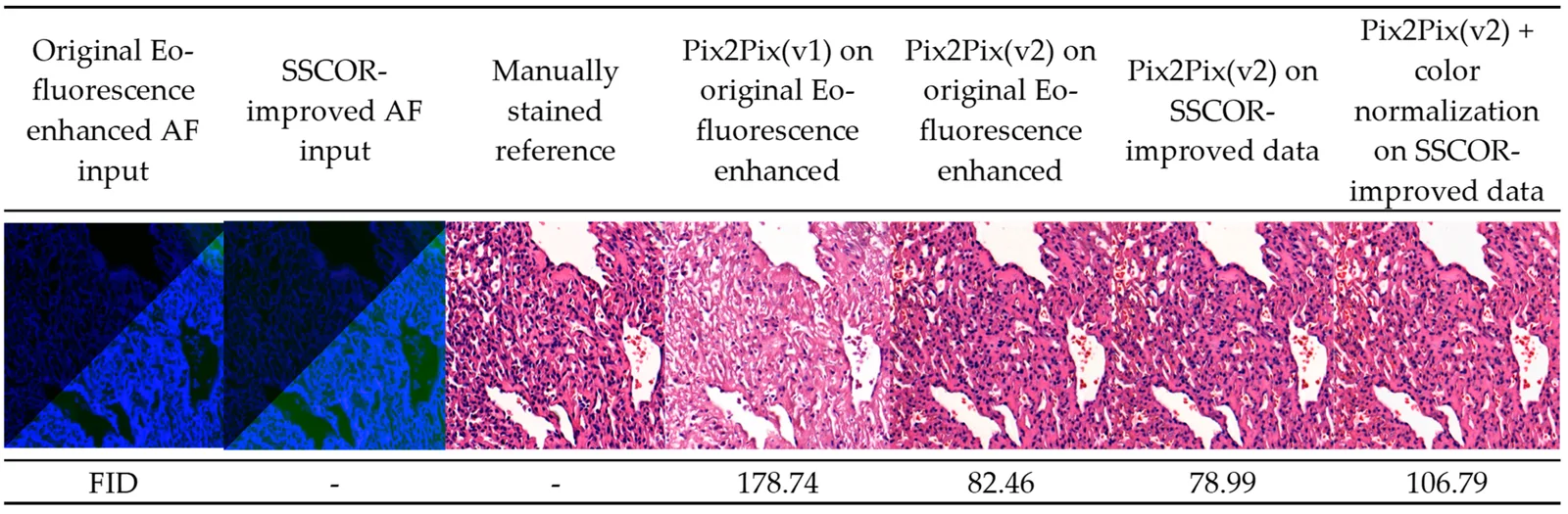
Staining results and FID score of different methods and inputs.

Figure 16 shows FID = 178.74 for Pix2Pix(v1) on the original input data and 82.46 for Pix2Pix(v2) on the original input data. Applying the same Pix2Pix(v2) model to SSCOR-processed inputs yielded the lowest observed FID, 78.99, whereas subsequent CycleGAN color normalization increased FID to 106.79. Because these values come from single model instances and were not supported by repeated retraining or inferential testing, they are interpreted as observed differences under the evaluated pipeline rather than statistical evidence of superiority.

FID was also calculated for the channel-specific Pix2Pix(v3) outputs. For this comparison, each DAPI, Cy3, or FITC input patch came from the registered fluorescence scan of an H&E-stained section and was paired with the same full H&E target from the corresponding tissue region. The channel-wise models therefore share a common H&E reference domain, enabling a matched comparison of their generated feature distributions while keeping in mind that FID is not a channel-specific measure of biological information.

**Figure 17:**
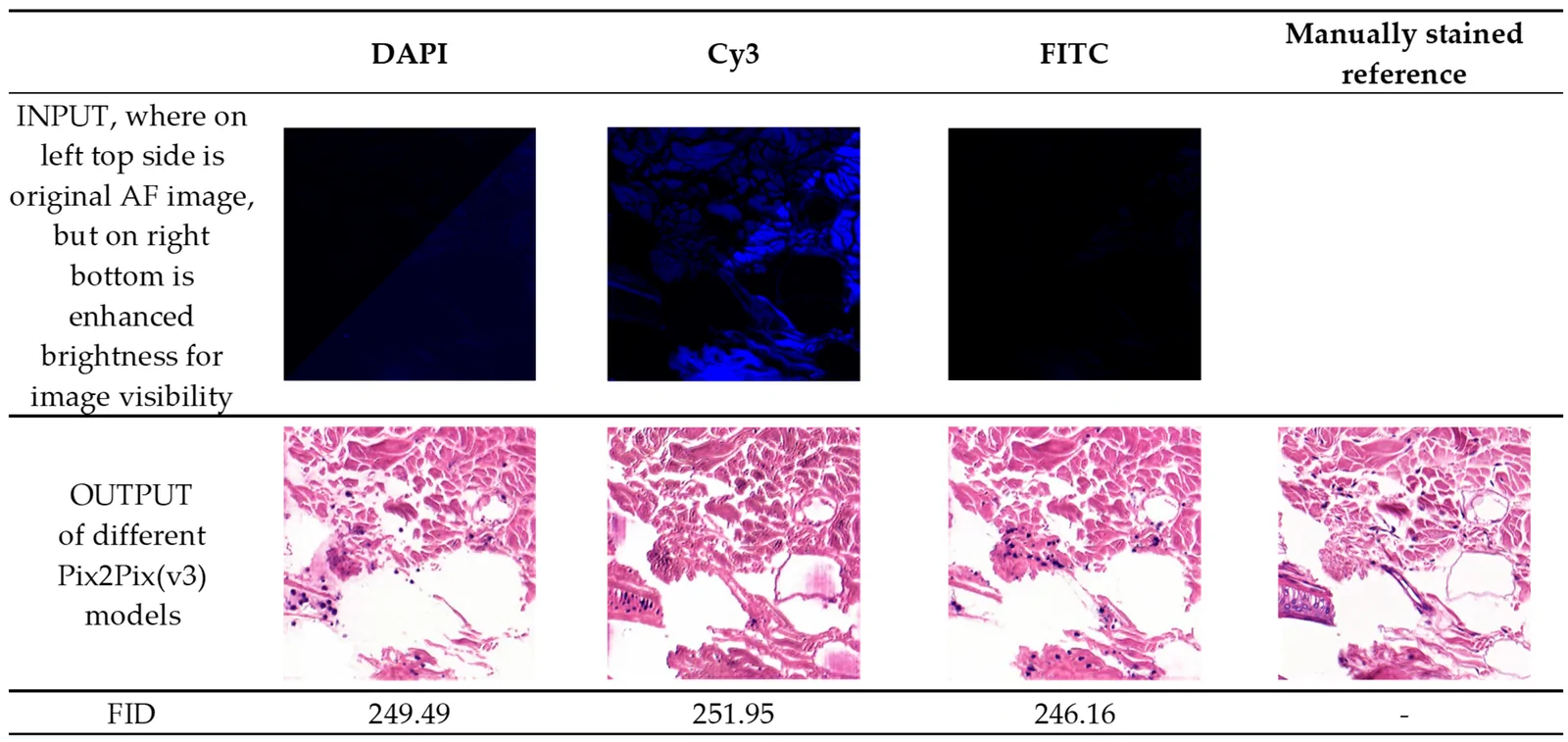
Channel-specific Pix2Pix(v3) outputs and FID values after transfer learning on registered single-channel fluorescence–H&E pairs.

Figure 17 shows that the transfer-learned single-channel models retained high FID values relative to the H&E reference (DAPI 249.49, Cy3 251.95, FITC 246.16), indicating substantial distribution-level mismatch. The similarity of these values means that FID did not clearly separate the three single-channel models. It also does not imply that a multi-channel input is required: in the four-case pathology pilot, the combined DAPI + Cy3 + FITC configuration scored lower than the DAPI-only configuration.

**Figure 18:**
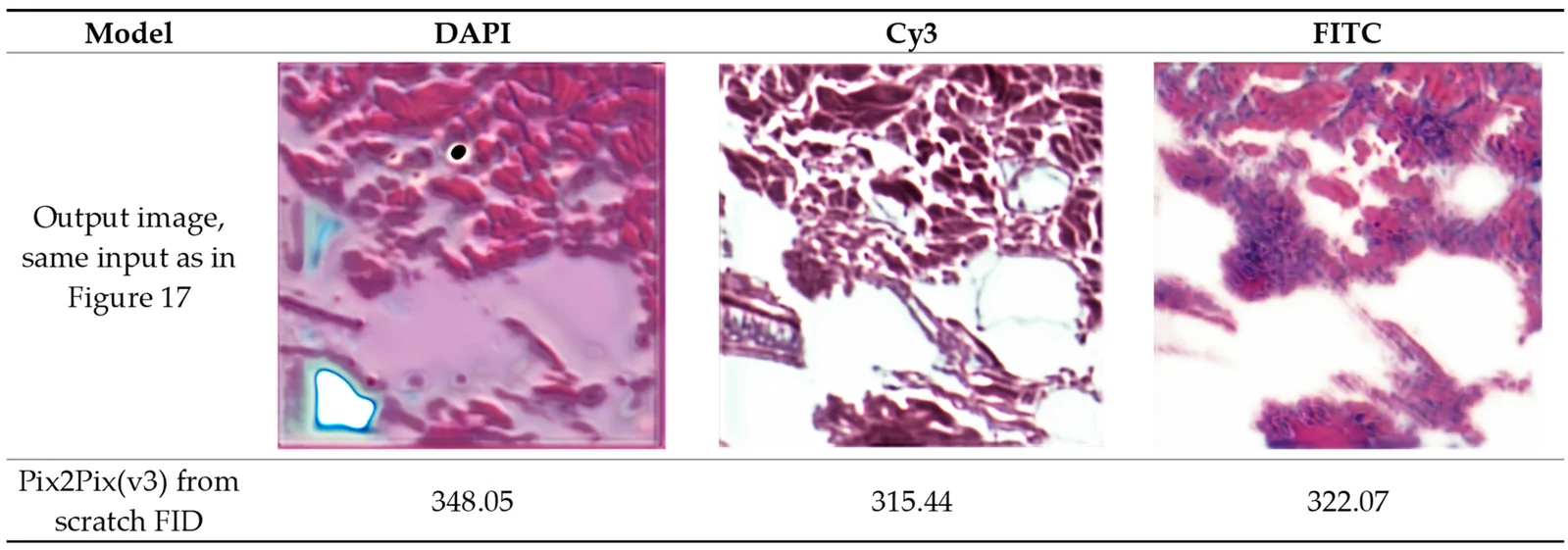
FID values for channel-specific Pix2Pix(v3) models trained from random initialization using the same held-out evaluation patches as the transfer-learning models.

For models trained from random initialization, FID values were 348.05 for DAPI, 315.44 for Cy3, and 322.07 for FITC (Figure 18). The transfer-learned and from-scratch models used identical held-out input patches and the same H&E reference set. Under this matched single-split evaluation, pretrained initialization was associated with lower observed FID for all three channels: 249.49 vs. 348.05 (DAPI), 251.95 vs. 315.44 (Cy3), and 246.16 vs. 322.07 (FITC), corresponding to reductions of 28.3%, 20.1%, and 23.6%, respectively. These values describe the observed runs and should not be interpreted as inferential evidence across independently retrained models.

### 4.4. SSIM for Pix2Pix Model

**Figure 19:**
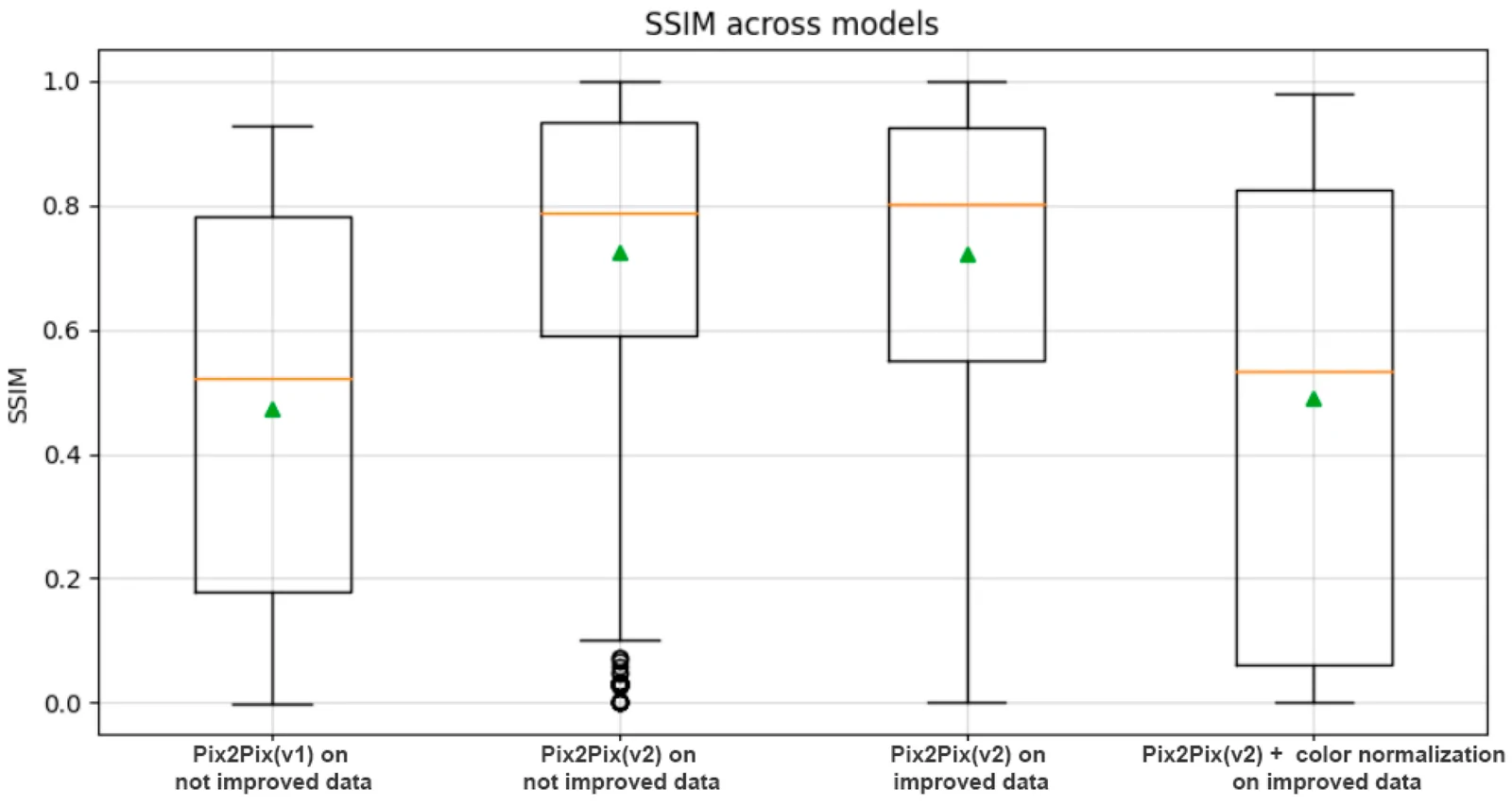
Structural Similarity Index (SSIM) results in boxplot representation.

Structural Similarity Index (SSIM) was analyzed for the four evaluated model/pipeline variants (Figure 19). Pix2Pix(v1) on the original inputs showed mean and median values around 0.45–0.50 and included very low outliers. Pix2Pix(v2) on the original and SSCOR-processed inputs showed higher observed SSIM, with mean values of approximately 0.72–0.73 and medians around 0.80. The SSCOR condition showed a narrower distribution in this evaluation, whereas Pix2Pix(v2) followed by CycleGAN color normalization showed lower SSIM, with a median around 0.52–0.55 and a wider distribution. SSIM measures image-level luminance, contrast, and structural similarity and should not be interpreted as proof of cellular or diagnostic fidelity. Accordingly, these results indicate higher observed structural similarity for Pix2Pix(v2) without CycleGAN normalization under the tested conditions, rather than formal statistical superiority.

### 4.5. Comparison of AF Channel and Source Image Format for TL-Generated Virtual Slides

**Figure 20:**
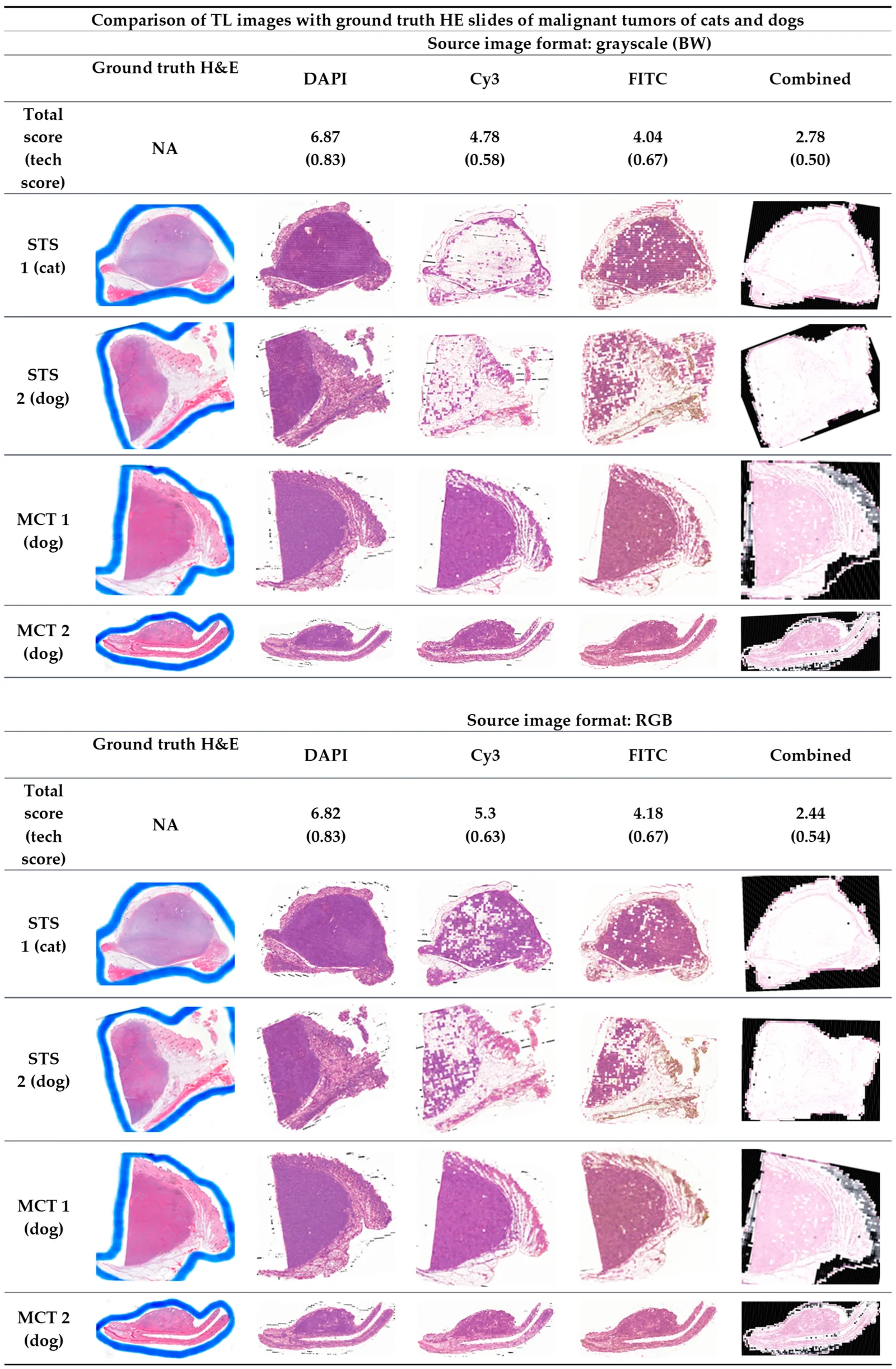
Effect of autofluorescence channel input and source image format on transfer learning-generated virtual H&E slides. Selected malignant tumors from cats and dogs (2 soft tissue sarcomas—STS, and mast cell tumor—MCT) are shown as ground truth H&E alongside virtual H&E outputs generated using single-channel autofluorescence inputs (DAPI, Cy3, or FITC) or a combined DAPI + Cy3 + FITC input. Total score for each input shows average for all 4 slides (max value 13); technical quality score shows average for all 4 slides (max value 1). Blue marker lines demarcate the area of tissue section scanning.

Within the four-case pilot TL-generated virtual H&E slides, visual output quality differed by both AF channel input and source image format (grayscale vs. RGB) (Figure 20). Across the evaluated channel conditions (DAPI, Cy3, FITC, and a combined DAPI + Cy3 + FITC input), DAPI-based inputs generated the most coherent virtual H&E appearance, with the best preservation of section architecture and tissue-wide contrast in STS and MCT of cats and dogs.

Cy3-based inputs resulted in marked discontinuities in tissue representation, characterized by pronounced tissue dropout for STS but not for MCT. This artifact was most evident when Cy3 was provided in grayscale source format, whereas RGB source format reduced—but did not eliminate—dropout. FITC-based inputs produced virtual slides with altered color rendition compared to ground truth H&E, introducing a brown tinge rather than the expected hematoxylin–eosin-like purple and red hues.

The poorest performance was observed with the combined DAPI + Cy3 + FITC input in both grayscale and RGB source formats. In particular, for STS this setting yielded very faint, low-contrast tissue representation that was not suitable for diagnostic interpretation at the whole-slide level.

**Figure 21:**
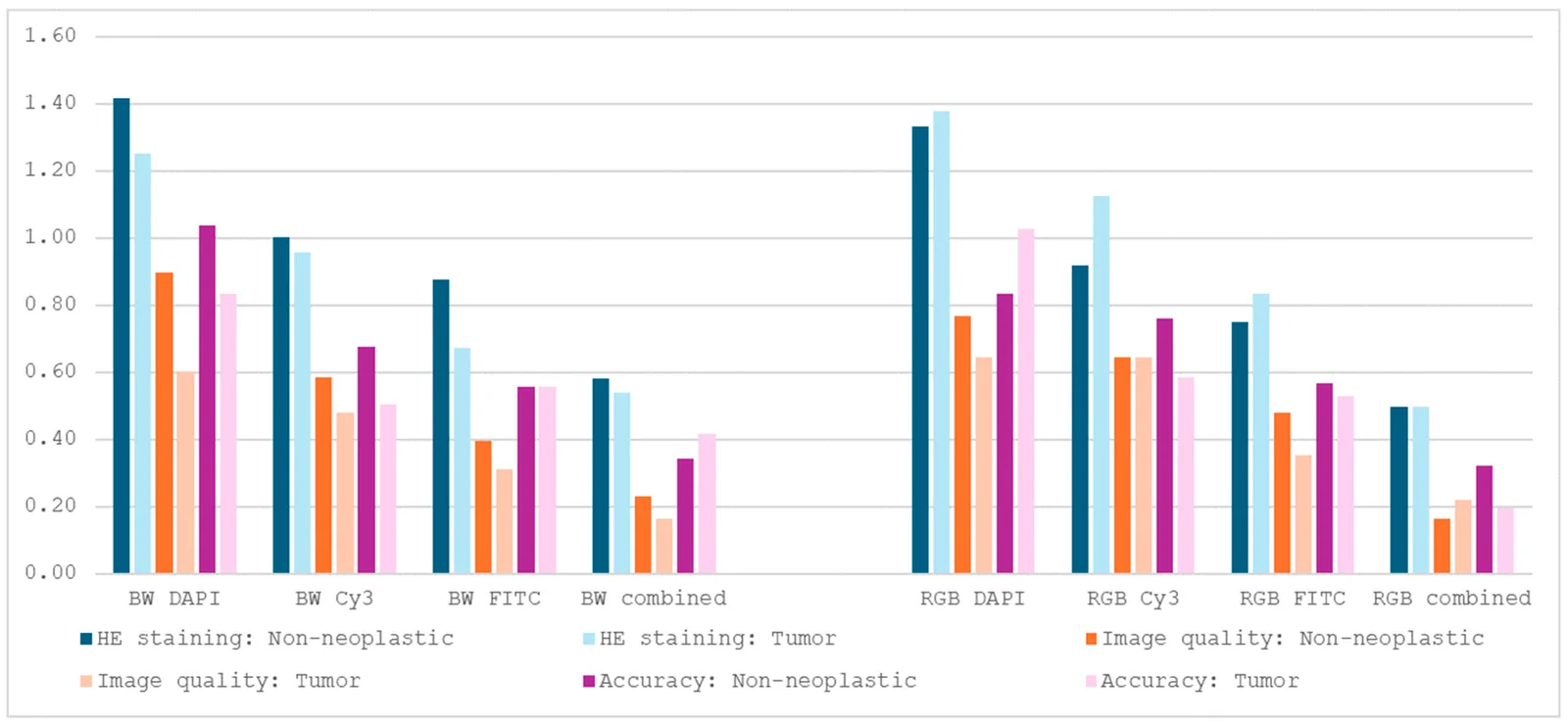
Composite sub-scores for assessment of quality of virtual staining slides generated via transfer learning method. BW = grayscale. For sub-score calculations see scoring system Table 1.

Composite sub-scores for virtual H&E slide quality assessment varied by category, tissue compartment, AF channel input, and source image format (Figure 21). Across all eight VS configurations (DAPI, Cy3, FITC, and combined DAPI + Cy3 + FITC in grayscale and RGB source formats), the highest composite sub-scores were consistently observed for H&E staining quality in both tumor and non-neoplastic tissues. In contrast, composite sub-scores for image quality and accuracy were uniformly lower across all configurations; when comparing these two categories, accuracy was generally slightly higher than image quality.

Within the four pilot cases, DAPI-based configurations achieved the highest overall composite scores, with the most favorable balance across categories. Composite profiles for grayscale DAPI and RGB DAPI were highly similar, with the primary difference being compartment-specific accuracy: grayscale DAPI yielded higher accuracy in non-neoplastic tissues, whereas RGB DAPI yielded higher accuracy in tumor tissues. Cy3- and FITC-based configurations showed intermediate composite sub-scores, and the combined DAPI + Cy3 + FITC configuration consistently performed worst in both grayscale and RGB formats, aligning with the qualitatively faint/low-information whole-slide appearance observed in representative cases.

When comparing source image formats, RGB format modestly enhanced composite sub-scores for Cy3, FITC, and the combined input relative to grayscale, supporting a small but consistent benefit of RGB outside the DAPI-only setting.

Across all eight VS configurations, image quality and accuracy at the cellular level were consistently limited. In particular, cellular and nuclear details were poorly preserved, precluding reliable assessment of fine cytomorphologic features required for tumor recognition, evaluation, and grading. Consequently, the virtual slides generated via TL method were not suitable for interpretation of features that depend on crisp cytoplasmic and nuclear contours, cellular and chromatin details, or individual inflammatory cell identification.

**Figure 22:**
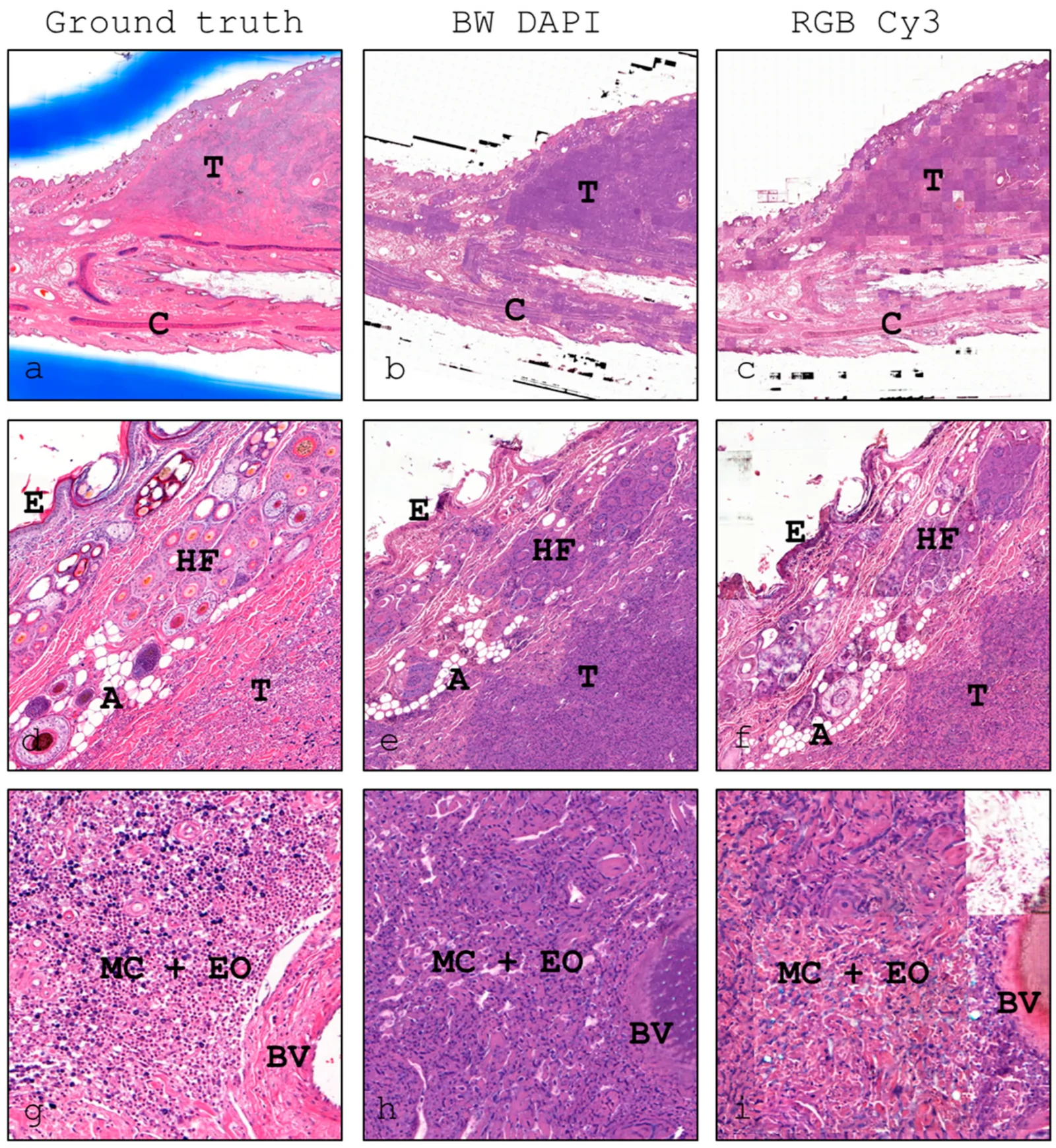
Mast cell tumor on an ear of a dog (#1542). Comparison between ground truth H&E stained slide **a**,**d**,**g**), grayscale (BW) DAPI (**b**,**e**,**h**) and RGB Cy3 (**c**,**f**,**i**) preparations of virtually stained slides (VS) using transfer learning method. Total slide score for grayscale DAPI is 6.44 (TQ = 0.83; NN = 3.33; T = 2.28); total slide score for RGB Cy3 is 6.86 (TQ = 0.67; NN = 3.44; T = 2.75) (TQ—technical quality; NN—non-neoplastic tissues, T—tumor tissues). At low magnification, both VS preparations maintain overall section architecture and allow recognition of tumor and major background tissues; however, at higher magnification the limitations in cytologic fidelity become apparent. In the grayscale DAPI output, mast cells and eosinophils cannot be distinctly recognized, consistent with the overall loss of fine cellular detail (**h**). In the RGB Cy3 output (**i**), these cellular populations are somewhat better preserved, but the image exhibits pronounced pixelation/tiling artifacts, which continues to limit confident cytologic interpretation. Image labels: T—tumor, C—cartilage, HF—hair follicles, A—adipose tissue, MC + EO—masts cells and eosinophils, BV—blood vessel.

Despite these limitations, certain configurations retained useful tissue-level fidelity. DAPI-based virtual slides consistently preserved larger-scale architecture, allowing recognition of broad anatomic landmarks and tissue interfaces, including tumor margins and adjacent non-neoplastic structures (notably subcutis, skeletal muscle, and adnexal structures such as hair follicles). A similar preservation of larger structures was also observed in MCT generated using the RGB Cy3 configuration, in which tumor distribution and relationships to surrounding tissues remained appreciable (Figure 22). Interestingly, RGB Cy3 was the only configuration that produced a slightly better image of eosinophils in addition to mast cells. However, equal tissue quality was not observed in RGB Cy3 STS virtually stained slides; therefore, its overall pilot score was lower than that of DAPI.

### 4.6. Quality and Accuracy of TL Virtual H&E in Clinical Veterinary Specimens

The pilot-selected transfer learning configuration (grayscale DAPI) was applied to 25 clinical malignant tumors from cats and dogs, comprising 13 STS and 12 MCT. Virtually stained H&E slides were evaluated semiquantitatively across three categories (H&E staining, image quality, accuracy), each recorded separately for non-neoplastic tissues and tumor tissues, and complemented by a technical quality score (section-wide) reflecting section completeness and staining uniformity. Across this clinical cohort, total slide scores were moderate relative to the theoretical maximum (13), with numerically higher mean total scores in STS than MCT. For STS, the mean total slide score was 5.95 ± 1.06 (range 4.00–7.64), whereas for MCT, the mean total slide score was 5.33 ± 0.91 (range 3.88–6.49). Technical quality (TQ) score was similar across tumor types and showed a narrow range (STS: mean 0.78 ± 0.08, range 0.67–0.83; MCT: mean 0.79 ± 0.07, range 0.67–0.83), indicating that section-wide technical performance was broadly stable under the applied configuration, while other categories and tissue-specific fidelity accounted for most variability in total slide scores.

**Figure 23:**
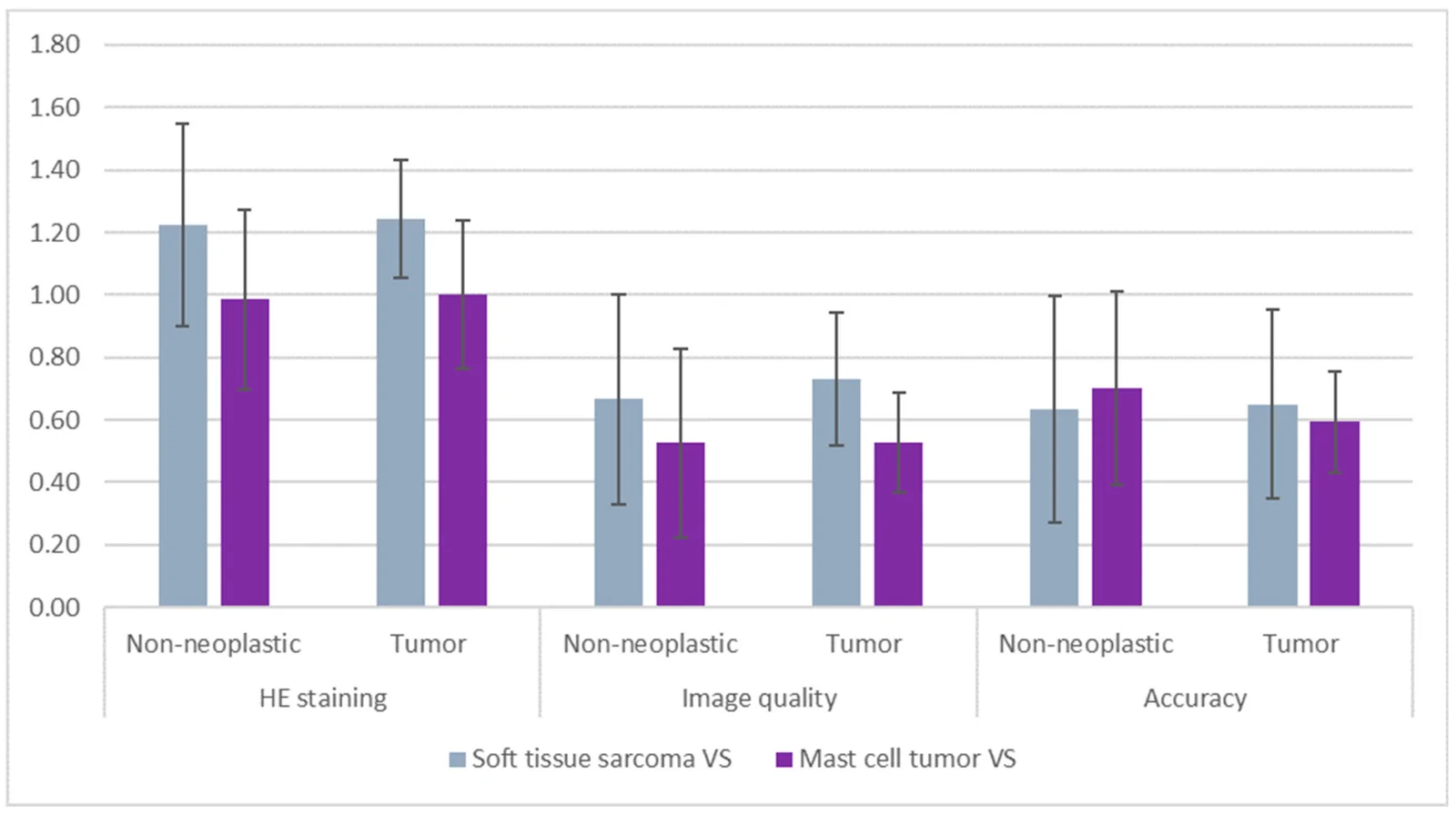
Comparative quality of transfer learning-generated virtually stained soft tissue sarcoma and mast cell tumor (composite sub-scores). VS—virtually stained slides; bars—standard deviation.

Across both tumor groups, the highest scores were consistently observed for H&E staining, whereas image quality and accuracy were lower, mirroring the observer impression that VS slides more reliably reproduced broad H&E-like staining patterns and tissue-level architecture than fine diagnostic detail (Figure 23).

Exploratory comparisons showed numerically higher scores in STS than MCT for several measures. The largest differences were observed for H&E staining quality in tumor tissue (mean ± SD: 1.24 ± 0.19 vs. 1.00 ± 0.24; mean difference 0.24; 95% CI 0.05–0.43; unadjusted *p* = 0.015) and image quality in tumor tissue (0.73 ± 0.21 vs. 0.53 ± 0.16; mean difference 0.20; 95% CI 0.04–0.36; unadjusted *p* = 0.015). However, neither difference remained statistically significant after Holm correction for multiple comparisons. The remaining compartment-specific comparisons likewise showed no evidence of statistically significant differences after correction. Given the small subgroup sizes, these comparisons were interpreted as exploratory and hypothesis-generating.

**Figure 24:**
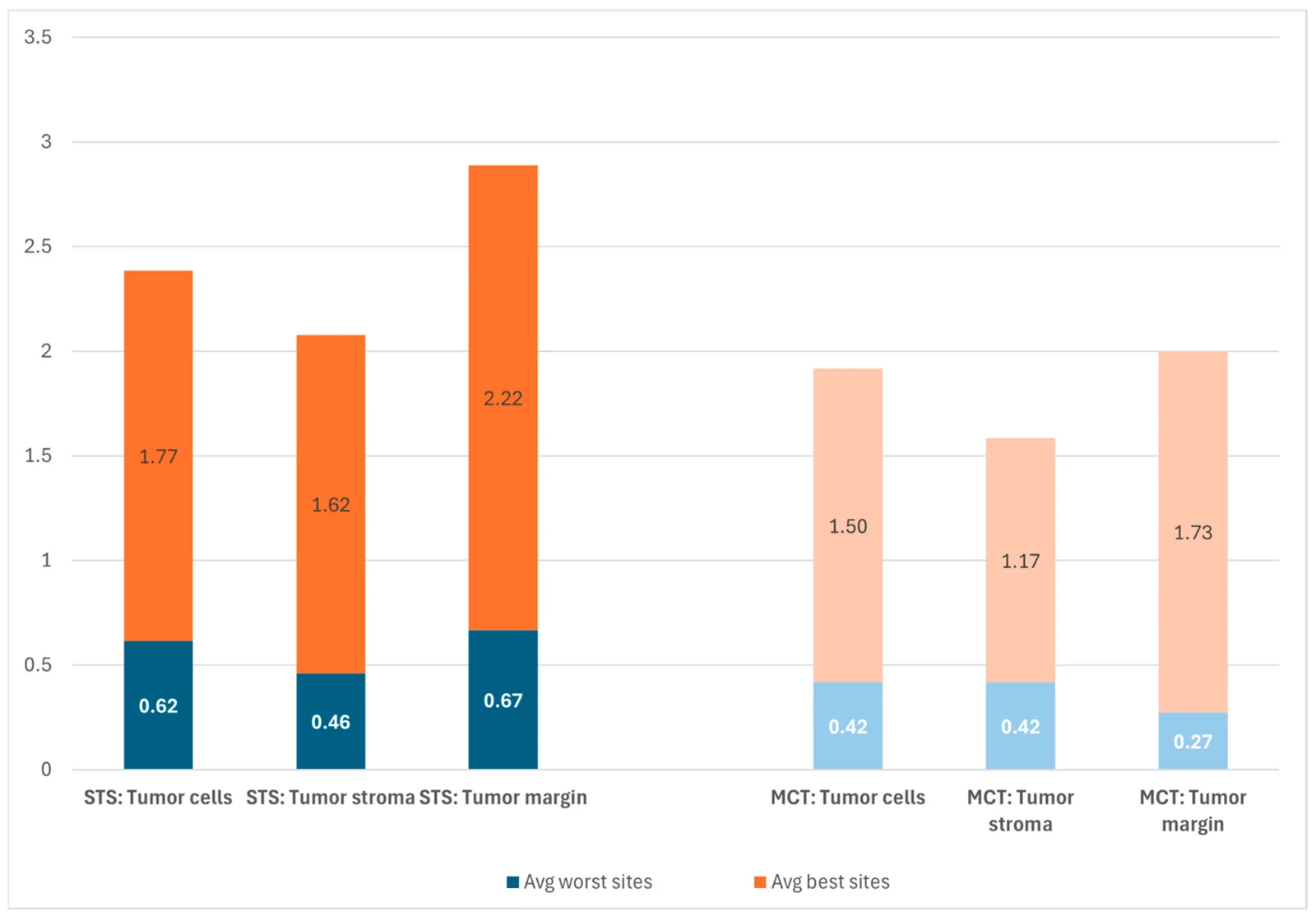
Average raw scores for accuracy in tumor depiction in virtual slides generated by transfer learning method. STS—soft tissue sarcoma; MCT—mast cell tumor.

**Figure 25:**
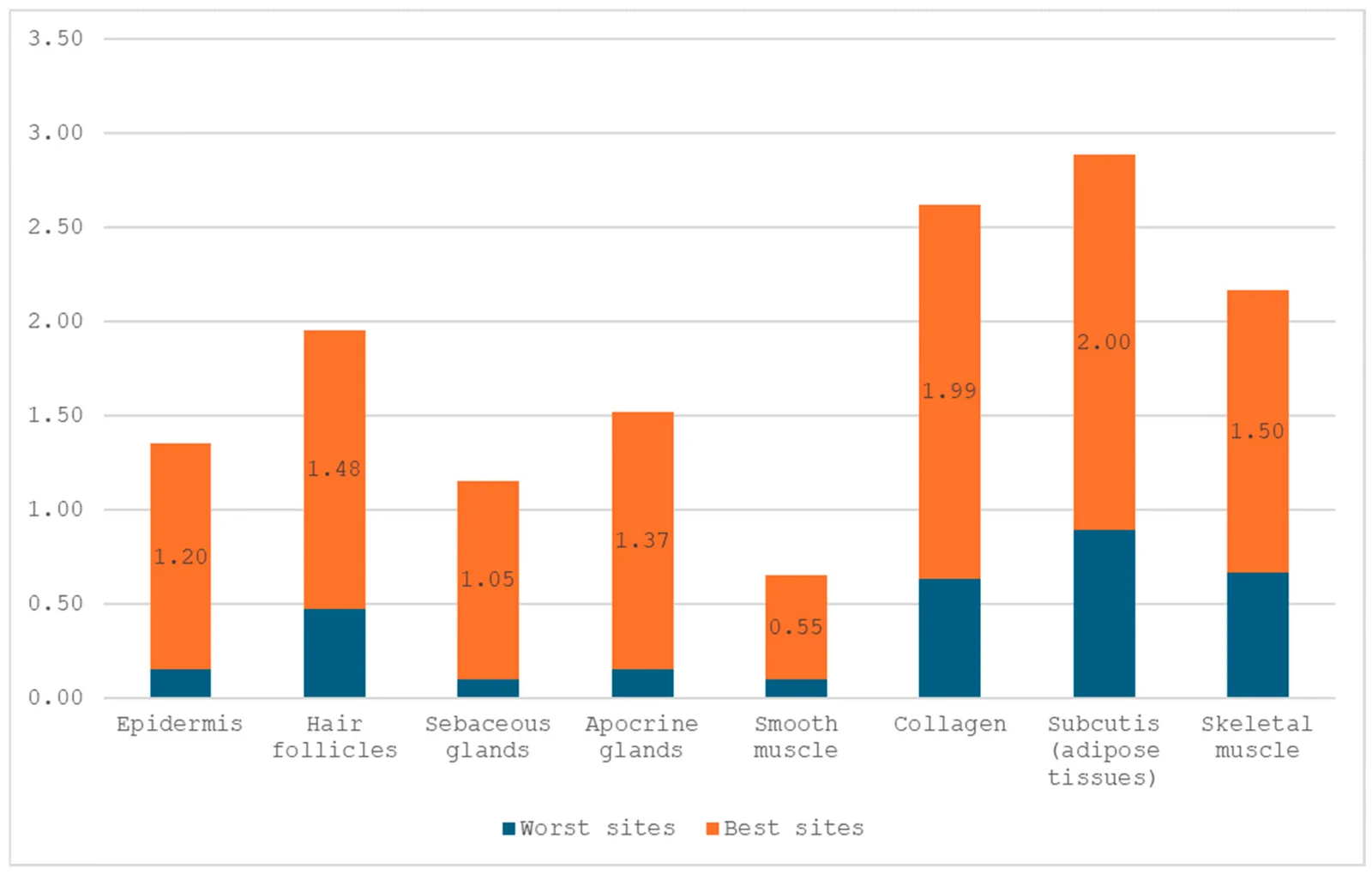
Average raw scores for accuracy in non-neoplastic tissue depiction in virtual slides generated by transfer learning method.

To better contextualize “accuracy” as a diagnostic construct, feature-level raw scores were summarized for tumor depiction and for key non-neoplastic tissues (Figure 24 and Figure 25). For tumor depiction, tumor margin accuracy tended to be higher than depiction of tumor cells or tumor stroma, particularly in STS. In STS, best-site averages were highest for tumor margins (2.22), followed by tumor cells (1.77) and tumor stroma (1.62). In MCT, best-site averages were lower overall and again showed relatively better performance for tumor margins (1.73) and tumor cells (1.50) than for tumor stroma (1.17). Across all tumor features and both tumor types, worst-site averages were substantially lower than best-site averages, indicating marked regional heterogeneity within slides.

For non-neoplastic tissues, accuracy similarly varied by tissue type (Figure 25). Collagen (best-site average 1.99) and subcutis/adipose tissue (2.00) tended to be among the most reliably depicted structures, followed by skeletal muscle (1.50). Hair follicles (1.48) and apocrine glands (1.37) were moderately well preserved, whereas epidermis (1.20) and sebaceous glands (1.05) were lower. Smooth muscle showed the lowest accuracy (0.55), consistent with limited preservation of fine structural detail in virtually stained slides.

**Figure 26:**
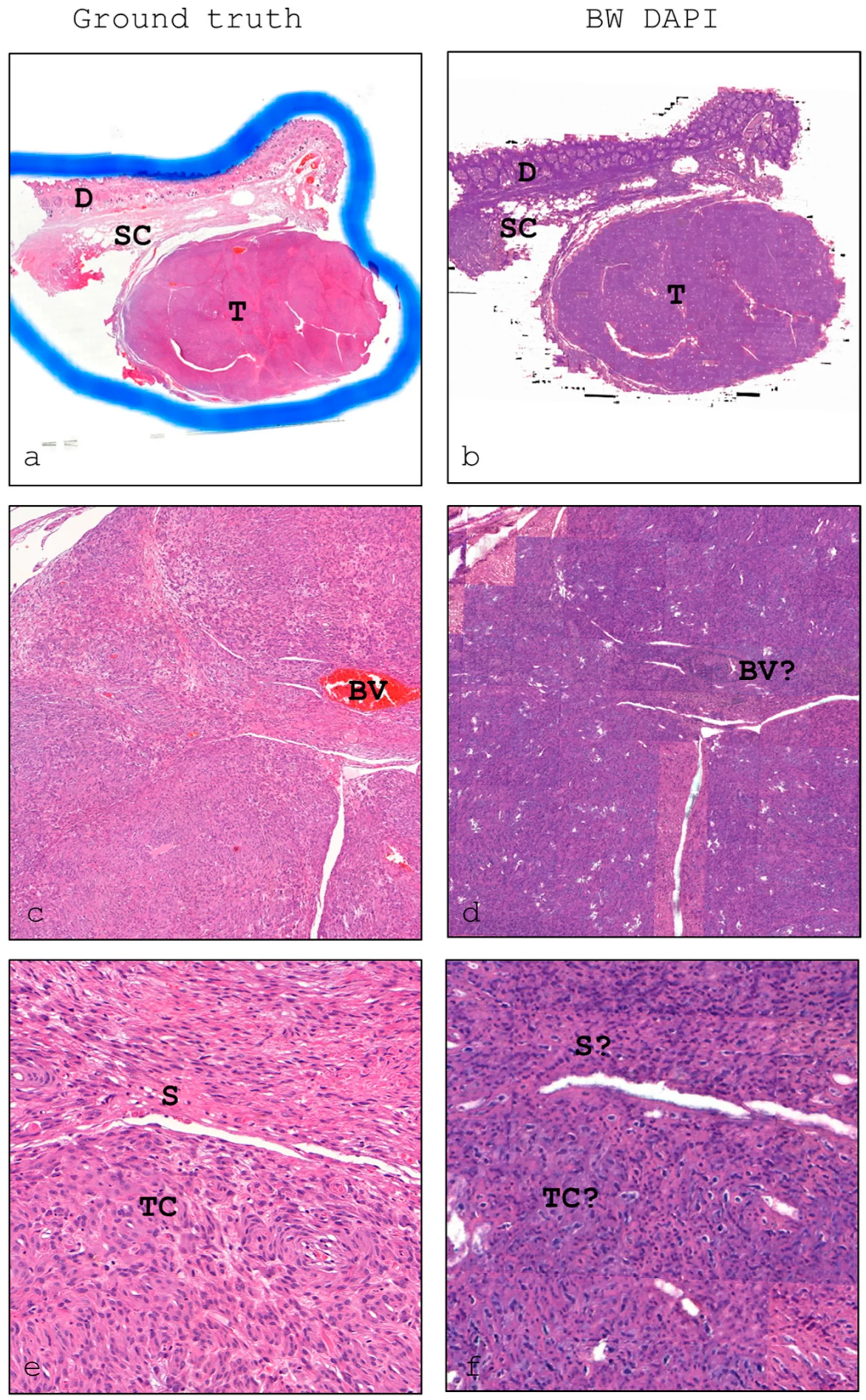
Soft tissue sarcoma in the subcutis of a dog (#1369). Comparison between ground truth H&E stained slide (**a**,**c**,**e**) and virtually stained slide (VS) using transfer learning method (grayscale DAPI) (**b**,**d**,**f**). Total slide score for virtual staining is 5.19 (TQ = 0.83; NN = 1.64; T = 2.72; TQ—technical quality; NN–non-neoplastic tissues; T—tumor tissues). At low magnification, overall section architecture and the distribution of tumor relative to surrounding dermis and subcutis were preserved (**b**). At higher magnification, distinction between tumor cells and supporting stroma was reduced, with blurring of cytomorphologic detail and loss of crisp nuclear features, limiting confident evaluation of fine diagnostic criteria (**f**). T—tumor, SC—subcutis, BV—blood vessel, BV?—blood vessel cannot be recognized, S—stroma; S?—stroma is not adequately represented; TC—tumor cells in bundles, TC?—tumor cells and their arrangement patterns are indistinct.

Across clinical specimens, several recurring limitations were noted that aligned with the quantitative profile of lower image quality and accuracy relative to H&E staining. Most prominently, cellular and nuclear detail was poorly preserved, limiting the assessment of fine cytomorphologic features used for tumor recognition, grading, and evaluation. In many slides, region-dependent artifacts were observed, including pixelation/tiling, localized loss of detail, and frequent ambiguity in discriminating tumor cells versus stroma or interpreting delicate structures (e.g., small vessels) (Figure 26).

From a diagnostic perspective, these limitations were evident in both tumor types. In STS, VS often did not accurately reflect cellularity and tumor cell–stroma relationships: stromal areas appeared falsely cellular (with spurious nuclear-like detail), while tumor stroma itself was underrepresented, obscuring fascicular/bundled tumor cell patterns and stromal composition. Tumor-associated features including mucinous matrix, necrosis, and inflammation were not reproduced. In MCT, mast cell granularity was not represented and eosinophils were not identifiable, limiting morphologic confirmation. Overall, these shortcomings indicate that, at the current stage, transfer learning-generated virtual slides are not sufficient for confident, specific tumor diagnosis, although they generally preserve subgross tumor distribution and can support assessment of tumor margins when margins are well delineated on the ground truth H&E.

## 5. Discussion

This study demonstrated a practical two-stage transfer-learning route for generating H&E-like virtual staining from native label-free AF WSIs when direct paired native AF–H&E supervision was not available. The data-engineering pipeline intentionally separated two domains: (i) same-section fluorescence images of H&E-stained tissue paired with brightfield H&E for supervised model development, and (ii) native AF images from adjacent unstained sections used as the target domain for inference. Registration, tiling, channel extraction, and artifact preprocessing therefore directly determined which information could be used for supervised learning and which remained unpaired at deployment. Pathology evaluation showed that the resulting VS preserved tissue-level architecture and often tumor distribution and margins, but not sufficient cellular and nuclear detail for confident diagnosis or grading. In the four-case pilot, DAPI was the most informative single-channel configuration among those tested. On an NVIDIA A100 GPU (40 GB VRAM), inference for a 512 × 512-pixel tile required approximately 3 s; slide #489he (260 tiles) required 13 min (780 s), corresponding to slightly under 15 s/mm^2^ based on an estimated tile area of ~0.22 mm^2^. Four-channel AF acquisition required approximately 30–60 min per slide depending on tissue area, whereas a single DAPI scan required approximately 15 min.

A central methodological point is that the workflow uses transfer learning rather than semi-supervised optimization on the unpaired target data. Stage 1 uses supervised same-section fluorescence–H&E pairs. Stage 2 again uses supervised channel-wise fluorescence–H&E pairs to adapt Pix2Pix(v2) into DAPI-, Cy3-, and FITC-specific Pix2Pix(v3) models. Native AF images lacking paired H&E are introduced only during inference. Transfer is therefore mediated by pretrained parameters and channel-specific fine-tuning, without an additional morphology- or color-specific supervisory loss.

On the engineering side, iterative model and pipeline development identified specific factors that changed VS output quality. Replacing the baseline generator with a Dense U-Net, revising effective patch resolution, and fine-tuning on a curated paired subset reduced incomplete or blank regions in the tested outputs. By contrast, CycleGAN-based post-processing altered the H&E color distribution but reduced SSIM, increased FID, and received poorer expert scores under the tested configuration. Color harmonization was therefore not retained as part of the final workflow.

Across the engineering experiments, the largest gains were associated with revising the Pix2Pix generator and training data and with transfer learning, whereas post-hoc color normalization did not improve the evaluated outputs. Pix2Pix(v2) showed lower FID and higher SSIM than Pix2Pix(v1) under the tested conditions. Because the same fixed Pix2Pix(v2) model was applied to raw and SSCOR-processed inputs, differences between those conditions reflect preprocessing-induced domain shift rather than retraining. CycleGAN normalization further changed the output distribution and, under the tested configuration, increased FID and reduced SSIM and expert scores. For target-domain native AF, direct Pix2Pix(v2) inference on isolated channels often produced incomplete outputs, whereas channel-specific Pix2Pix(v3) fine-tuning produced more complete stains. However, the single-channel Pix2Pix(v3) FID values remained high, showing that transfer learning reduced but did not eliminate the distribution gap to real H&E. These FID results do not show that multi-channel input is superior; the combined DAPI + Cy3 + FITC configuration underperformed relative to the DAPI-only configuration in the four-case pilot. Overall, the results emphasize domain matching, structural information, and validation at the intended WSI scale rather than color uniformity alone.

The high Pix2Pix(v3) FID values can be explained by several nonexclusive factors. First, a single fluorescence channel is an information bottleneck: morphology weakly represented in that channel cannot be uniquely reconstructed from the input. Second, the channel-specific model is supervised against the full H&E target, so the target contains structures and stain components that may have little or no corresponding contrast in a given input band. Third, the source-domain channel pairs were derived from fluorescence scans of H&E-stained sections, whereas deployment used native AF from unstained sections, introducing an additional source-to-target domain shift. Finally, Cy5 was unavailable for channel-specific training because of the export limitation. The residual mismatch should therefore be interpreted as the combined effect of limited single-channel information, cross-domain transfer, and model optimization rather than as failure of one isolated component.

FID and SSIM were used as descriptive image-quality metrics. FID relies on generic Inception-v3 features rather than pathology-specific representations, while SSIM measures image-level luminance, contrast, and structure and does not establish diagnostic fidelity. In addition, the transfer-learning versus from-scratch comparison used a single patch-level 80/20 split and single trained instances rather than repeated slide-level cross-validation. Consequently, reported numerical differences should be interpreted as observed performance under the evaluated split, not as statistically established model superiority. Independent case-level splits, repeated retraining, and pathology-specific perceptual or feature metrics are appropriate next steps.

The study also clarified why direct supervised training on native AF–H&E pairs was not feasible. Same-section registration was available for the source domain because fluorescence and brightfield H&E were acquired from the same H&E-stained physical section. In contrast, native label-free AF was acquired from the adjacent unstained serial section, whose tissue geometry is not identical to the H&E-stained section; a global registration cannot create true one-to-one cellular correspondence where biological structures differ between sections. For this reason, native AF was not treated as a valid pixel-paired training target. Future same-section acquisition of label-free AF followed by H&E staining and reimaging would provide a stronger basis for direct supervised AF→H&E learning and independent paired validation.

Whole-slide processing constraints further shaped attainable resolution and, consequently, VS quality. Creation of paired datasets and scalable preprocessing were limited by the proprietary .mrxs WSI format, fragile container structure, and the need for conversion to .tiff to support reproducible workflows. The converted data did not preserve an efficient multi-resolution pyramidal structure for memory-efficient random access, so high-magnification extraction required loading large pixel regions into RAM and was prone to memory-related interruptions. Under these constraints, reliable extraction was limited to approximately 4× magnification, directly restricting the fine structural information available for learning. Vendor export also limited channel extraction to three channels despite four being acquired. Despite these limitations, the pipeline (HSV masking; ORB descriptor matching; RANSAC homography estimation; multi-resolution tiling; SSCOR preprocessing) enabled construction of 14 datasets (>272 WSIs) and successful same-section fluorescence–H&E registration of 60 WSIs for source-domain training and evaluation.

A consistent technical finding was that VS quality depended strongly on the AF input representation and on how channels were encoded. In the four-case pilot comparison across channels and source formats, DAPI-based inputs achieved the highest overall scores and produced the most coherent whole-slide VS within the tested cases. In contrast, Cy3-based inputs produced discontinuities and tissue dropout in some settings, and FITC-based inputs often yielded altered color rendition (e.g., brown tinge rather than expected hematoxylin–eosin balance). The combined DAPI + Cy3 + FITC configuration consistently performed worst and could yield faint, low-contrast tissue representations that were not diagnostically useful. Beyond channel choice, Pix2Pix outputs also differed when the same channel was provided as grayscale versus RGB-encoded representations, consistent with sensitivity to the numerical tensor structure rather than a simple “cosmetic” color change. Collectively, these results argued against assuming that more channels or color encodings will necessarily improve performance and supported systematic evaluation of encoding/normalization strategies as part of future optimization. Because configuration selection was based on only four cases, the apparent advantage of grayscale DAPI should be interpreted as a pilot finding that may be sample-dependent rather than as a universally optimal channel choice. Because the same overall cohort framework informed subsequent evaluation, optimistic selection bias cannot be excluded; independent case-level or nested cross-validation is required.

Regarding output fidelity, VS images remained inferior to manually stained H&E due to imperfect translation and AI-related artifacts. Nevertheless, two improvement directions can be proposed. First, higher-quality input acquisition—particularly increased scanning resolution and optical sharpness (e.g., higher numerical aperture objectives and/or higher magnification capture, ideally integrated into conventional slide-scanner workflows)—is expected to provide more structural detail during training and thereby improve VS fidelity; however, such implementation would result in significantly increased slide scanning time. Second, an additional neural network can be applied to enhance perceived detail (e.g., learned upscaling or super-resolution). This approach may introduce additional artifacts and therefore requires careful validation; however, related strategies have been explored previously [37,38].

From the pathology perspective, a major contribution was the semiquantitative scoring system that integrated technical quality, H&E staining quality, image quality, and accuracy, and explicitly captured best and worst regions in both non-neoplastic and tumor compartments. This structure addressed a recurring characteristic of VS—marked within-slide heterogeneity—where acceptable tissue-level appearance could coexist with localized dropout, tiling artifacts, pixelation, or loss of detail. By converting these observations into numerical values, the scoring framework enabled slide-to-slide comparison and provided a practical method to quantify improvements during iterative optimization. At the same time, because scoring was performed by a single pathologist, the framework requires future multi-observer validation and interobserver agreement testing to support broader generalization. This limits the reproducibility and generalizability of pathology-facing conclusions in the present study.

In the clinical cohort (13 STS, 12 MCT), total slide scores were moderate relative to the theoretical maximum, and H&E-like staining tended to score higher than image quality and accuracy. Exploratory single-observer comparisons showed numerically higher H&E staining-quality and image-quality scores in STS than MCT tumor regions; however, these differences did not remain statistically significant after correction for multiple comparisons. Given the small subgroup sizes and absence of multi-observer validation, these patterns should be considered hypothesis-generating rather than evidence of an established tumor-type effect. Feature-level descriptive assessment nevertheless suggested that tumor margins were more reliably depicted than tumor cells and especially tumor stroma.

Morphologically, VS preserved overall section architecture and the distribution of tumor relative to surrounding tissues, and tumor margins were often recognizable—particularly when margins were well delineated on ground-truth H&E. In non-neoplastic tissues, collagen and subcutis/adipose tissue were among the most reliably depicted structures, with intermediate performance for skeletal muscle and adnexal structures, while smooth muscle of hair follicles showed the lowest accuracy. These strengths suggested that, even under current resolution constraints, the method retained value for section-wide orientation, recognition of major tissue compartments, and margin-level context in selected cases.

However, diagnostically critical limitations remained substantial. The dominant failure mode across tumor types was reduced sharpness and loss of cellular/nuclear detail, limiting assessment of cytomorphologic criteria required for confident tumor diagnosis and grading. Region-dependent artifacts (including pixelation/tiling and localized loss of detail) further reduced interpretability at higher magnification. These shortcomings manifested with tumor-specific patterns: in STS, VS often distorted tumor cell–stroma relationships, including falsely “cellular” stroma and underrepresentation of true tumor stroma, obscuring fascicular patterns and stromal composition; tumor-associated features such as mucinous matrix, necrosis, and inflammation were not reliably reproduced. In MCT, mast cell granularity was not convincingly represented and eosinophils were often not identifiable even when present in ground truth, limiting cytologic confirmation. Overall, TL-generated VS were therefore better suited to preserving tissue-level context and margin-related information than to reproducing the cytologic and stromal detail required for definitive diagnosis and grading at this stage.

In summary, our work showed that TL provided a practical route to VS generation from unpaired AF whole-slide material, reducing reliance on fully paired datasets and enabling translation under realistic acquisition constraints. Importantly, the results also underscored that VS methods—whether implemented with or without TL—must be tested and validated on clinical specimens, because performance can vary across tissue compartments and within slides. By evaluating two malignant tumor types from cats and dogs together with adjacent non-neoplastic tissues, this exploratory single-observer assessment suggested possible tumor-type-related differences in VS performance, emphasizing the need for diagnostic validation under real-world conditions. The proposed pipeline and scoring framework provide a measurement-based way to guide optimization and clinical validation, particularly by improving resolution and WSI handling, using targeted training for critical morphologies, and confirming performance with multi-observer assessment.

## 6. Conclusions

(1) Transfer learning enabled H&E-like VS inference on native, unstained AF sections for which paired H&E targets were unavailable. The approach did not eliminate paired supervision: Pix2Pix(v1/v2) and channel-specific Pix2Pix(v3) were trained or fine-tuned using registered fluorescence–H&E pairs from H&E-stained source sections, and the learned parameters were then transferred to native AF inference.

(2) In the four-case pilot, VS output quality varied substantially with AF input representation and encoding, and grayscale DAPI yielded the highest overall scores and the most coherent whole-slide appearance among the tested configurations. This pilot finding requires confirmation using independent cases or case-level/nested cross-validation.

(3) Whole-slide dataset construction and high-magnification processing were constrained by WSI format and memory limitations, restricting reliable extraction to approximately 4× magnification and shaping attainable structural fidelity.

(4) The diagnostically oriented scoring framework provided a structured semiquantitative approach for capturing within-slide heterogeneity and comparing slides during method optimization; however, its present application was limited to a single observer and requires multi-observer validation and assessment of interobserver agreement.

(5) In a clinical cohort of canine and feline STS and MCT, VS preserved tissue-level architecture and frequently allowed subgross tumor localization and margin recognition, but fine cytologic detail and diagnostically critical features were not reproduced sufficiently for confident tumor diagnosis or grading.

(6) VS performance was regionally heterogeneous, and exploratory single-observer comparisons suggested possible tumor-type-related differences; however, these differences were not statistically significant after correction for multiple comparisons. Larger independently validated cohorts and multi-observer assessment are required before drawing conclusions regarding tumor-type effects or routine diagnostic performance.

## Figures and Tables

**Table 1:** Scoring criteria for whole-slide images.

Category	Parameter	Score 0	Score 1	Score 2	Score 3
Technical quality (section-wide)	Uniformity of staining	Extremely patchy	Very patchy	Moderately patchy	Very uniform/slightly patchy
	Tissue section completeness	>50% missing	25–50% missing	5–25% missing	Full/<5% missing
H&E staining	H&E balance/color	One stain absent	Very poor	Moderately suboptimal	Optimal/slightly suboptimal
	Intensity/contrast	Extremely pale/dark	Severely suboptimal	Moderately suboptimal	Optimal/slightly suboptimal
Image quality	Sharpness	Completely blurry	Very blurry	Moderately blurry	Clear/slightly blurry
	Cellular details	None	Very poor	Moderately suboptimal	Optimal/slightly suboptimal
	Nuclear details	None	Very poor	Moderately suboptimal	Optimal/slightly suboptimal
	Non-cellular elements	Completely indistinct	Very indistinct	Moderately indistinct	Distinct/slightly indistinct
Accuracy	Tissue identification	Cannot identify	Barely identifiable	Identifiable but suboptimal	Perfect/nearly perfect

## Data Availability

Data and code supporting the findings of this study are available upon request from the authors.

## Supplementary Materials

The following supporting information can be downloaded at https://www.mdpi.com/article/10.3390/jimaging12100490/s1, Figure S1: Pix2Pix improvement for enhanced cellular detail.

## Abbreviations

The following abbreviations are used in this manuscript:

H&E	hematoxylin and eosin
DL	deep learning
VS	virtually staining
AI	artificial intelligence
AF	autofluorescence
NN	neural network
TL	transfer learning
STS	soft-tissue sarcomas
MCT	mast cell tumors
SSIM	Structural Similarity Index
SSCOR	Stripe Self-CORrection
FID	Fréchet Inception Distance
FFPE	formalin-fixed, paraffin-embedded
HSV	Hue Saturation Value
RANSAC	RAndom SAmple Consensus
WSI	whole-slide image
CUT	Contrastive Unpaired Translation
GAN	Generative Adversarial Network
ORB	Oriented FAST and Rotated BRIEF
ROI	region of interest
