# Supplementary material for: Assessment of Transfer Learning for H&E Virtual Staining in Veterinary Oncology
Source: J Imaging. 2026 Oct 6;12(10):490. doi: 10.3390/jimaging12100490 (PMC13705746; doi:10.3390/jimaging12100490)
Supplement: Supplementary file 1 [file jimaging-12-00490-s001.zip › jimaging-4507250-supplementary.pdf]

Journal of Imaging

MDPI

Supplementary Materials

Assessment of Transfer Learning for H&E Virtual Staining in Veterinary Oncology

Daira Viškere [1,2,*], Diāna Dupļevska [3], Ilze Matīse-VanHoutana [1,2], Romans Maļiks [3], Blaž Cugmas [1], Mindaugas Tamošiūnas [1] and Roberts Kadiķis [3,*]

[1] Biophotonics Laboratory, Institute of Atomic Physics and Spectroscopy, Faculty of Science and Technology, University of Latvia, 19 Raina Blvd., LV-1586 Riga, Latvia; imatise.vh@gmail.com (I.M.-V.H.); blaz.cugmas@lu.lv (B.C.); mindaugas.tamosiunas@lu.lv (M.T.)

[2] Faculty of Veterinary Medicine, Latvia University of Life Sciences and Technologies, 8 K. Helmana Str., LV-3004 Jelgava, Latvia

[3] Institute of Electronics and Computer Science, 14 Dzerbenes Str., LV-1006 Riga, Latvia; diana.duplevska@edi.lv (D.D.); romans.maliks@edi.lv (R.M.)

* Correspondence: daira.viskere@lbtu.lv (D.V.); roberts.kadikis@edi.lv (R.K.)

Autofluorescent	New 512x512 px H&E patch with higher resolution	Pix2Pix(v1) on higher resolution data	Pix2Pix(v2) on higher resolution data	Pix2Pix(v2) on SSCOR improved data	Pix2Pix(v2) on SSCOR improved data + stain norm
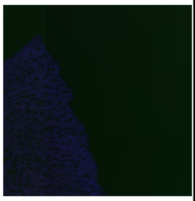	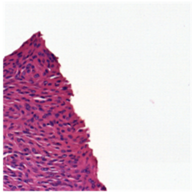	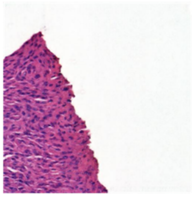			
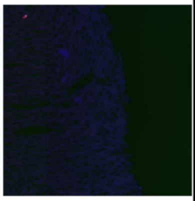	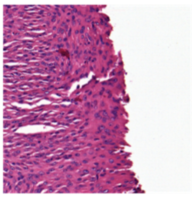	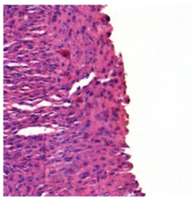			
FID		180.88			
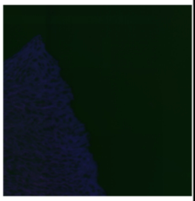	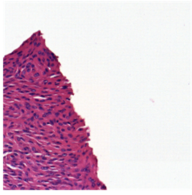	-	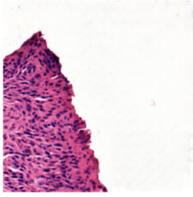	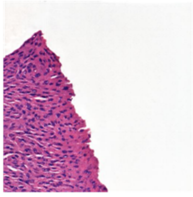	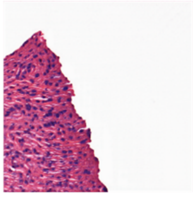
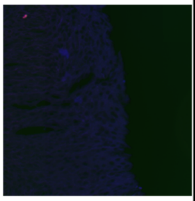	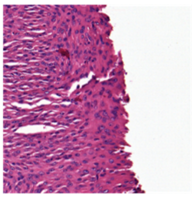	-	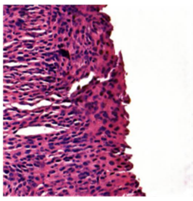	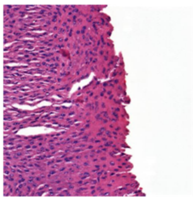	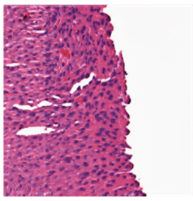
FID			152.77	78.99	106.79

Figure S1. Pix2Pix improvement for enhanced cellular detail.

 https://doi.org/10.3390/jimaging12100490

Raw autofluorescence (AF) images are first processed with the Pix2Pix(v1) model, trained on higher-resolution im-ages, which provides an initial virtual H&E output that still contains artifacts and missing regions. To improve re-construction quality, Pix2Pix(v1) was fine-tuned on a small, high-quality paired AF–H&E subset enriched for cel-lular detail, using random center-crop augmentation (factor 0.2–0.5) followed by resizing to 512 × 512 pixels. Man-ual early stopping was applied once the training losses reached a plateau, and checkpoint 46 was retained as Pix2Pix(v2). Pix2Pix(v2) was then applied without additional training to SSCOR-enhanced AF inputs; thus, SSCOR was evaluated as an input-preprocessing step while the Pix2Pix(v2) model remained fixed. Finally, a CycleGAN stain-normalization module was applied to the Pix2Pix(v2)-generated images to adapt their coloration toward a unified H&E reference style, reducing inter-slide variability.

https://doi.org/10.3390/jimaging12100490
